# Current developments of gene therapy in human diseases

**DOI:** 10.1002/mco2.645

**Published:** 2024-08-16

**Authors:** Fanfei Liu, Ruiting Li, Zilin Zhu, Yang Yang, Fang Lu

**Affiliations:** ^1^ Department of Ophthalmology West China Hospital Chengdu Sichuan China; ^2^ State Key Laboratory of Biotherapy West China Hospital Chengdu Sichuan China; ^3^ College of Life Sciences Sichuan University Chengdu Sichuan China

**Keywords:** AAV, clinical trials, CRISPR–Cas, gene therapy, human diseases

## Abstract

Gene therapy has witnessed substantial advancements in recent years, becoming a constructive tactic for treating various human diseases. This review presents a comprehensive overview of these developments, with a focus on their diverse applications in different disease contexts. It explores the evolution of gene delivery systems, encompassing viral (like adeno‐associated virus; AAV) and nonviral approaches, and evaluates their inherent strengths and limitations. Moreover, the review delves into the progress made in targeting specific tissues and cell types, spanning the eye, liver, muscles, and central nervous system, among others, using these gene technologies. This targeted approach is crucial in addressing a broad spectrum of genetic disorders, such as inherited lysosomal storage diseases, neurodegenerative disorders, and cardiovascular diseases. Recent clinical trials and successful outcomes in gene therapy, particularly those involving AAV and the clustered regularly interspaced short palindromic repeats (CRISPR)–CRISPR‐associated proteins, are highlighted, illuminating the transformative potentials of this approach in disease treatment. The review summarizes the current status of gene therapy, its prospects, and its capacity to significantly ameliorate patient outcomes and quality of life. By offering comprehensive analysis, this review provides invaluable insights for researchers, clinicians, and stakeholders, enriching the ongoing discourse on the trajectory of disease treatment.

## Graphical abstract

Gene therapy has seen remarkable advancements in recent decades, demonstrating its immense potential in treating a wide range of genetic and acquired diseases. Several AAV gene therapy products have been introduced to the market, addressing various conditions. This review explores the clinical applications of AAV in major human diseases, including ocular diseases, neuromuscular diseases, hematological diseases, neurological diseases, cardiovascular diseases, and lysosomal storage diseases.

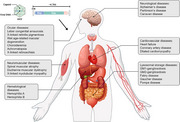

## 1 INTRODUCTION

Gene therapy, a groundbreaking strategy to treating human diseases, has seen remarkable advancements since its inception. This technique originated in the 1970s and involves adding, removing, or altering genetic materials within a patient's cells to mitigate or cure diseases.^1^ In recent decades, gene therapy has transitioned from a theoretical concept to a practical solution, with several therapies now approved for clinical use. Gene therapy encompasses various strategies such as gene replacement, silencing, addition, and editing utilizing viral or nonviral carriers to introduce exogenous nucleic acid(s) into target cells, thereby altering gene expression to correct or compensate for genetic defects and abnormalities.^2^ Gene replacement involves substituting a faulty gene with a healthy one, offering potential cures for numerous genetic disorders. Conversely, gene silencing aims to decrease or eliminate the activity of a specific harmful gene. Gene addition introduces a new gene into the genetic makeup of the host to combat diseases, whereas gene editing, perhaps the most advanced strategy, enables precise modification of the genetic code.^3^ Luxturna, the inaugural gene therapy authorized by the United States Food and Drug Administration (US FDA) in 2017, has demonstrated both safety and effectiveness in phase I/II clinical trials for treating Leber congenital amaurosis (LCA) type 2.^4^, ^5^, ^6^

Given the rapid pace of discoveries and clinical trials, it is crucial to offer a thorough analysis of the present status of gene therapy, including its applications, limitations, and prospects. By synthesizing existing literature, this review aims to offer a valuable resource for researchers, clinicians, and policymakers interested in understanding the latest developments and potential implications of gene therapy. This review covers several key aspects of gene therapy. First, it discusses various gene therapy approaches, including gene replacement, editing, and silencing, along with their underlying mechanisms and applications. Second, it highlights recent breakthroughs and success stories in gene therapy, such as the treatment of inherited genetic diseases like spinal muscular atrophy (SMA) and hemophilia. Additionally, it explores the challenges and limitations faced by gene therapy, including immune responses, off‐target effects, and ethical considerations. Furthermore, the review discusses future directions and emerging technologies in gene therapy, including the utilization of clustered regularly interspaced short palindromic repeats (CRISPR)–CRISPR‐associated protein (Cas)9 and viral vectors for targeted gene editing.

This review offers a comprehensive exploration of the current advancements in gene therapy for human diseases. It begins with an introduction to four genetic treatment modalities—gene replacement, silencing, addition, and editing—and proceeds to provide a perspective on gene therapy, including a detailed discussion on gene delivery systems. The application of gene therapy in various disease contexts is then explored, with a spotlight on recent clinical trials and successful outcomes. The paper culminates with a discussion on the prospects of gene therapy, offering invaluable insights into its potential as a transformative approach in healthcare. Throughout, the review maintains a logical and coherent sequence of information, thoroughly examining the current breakthroughs and challenges of gene therapy.

## 2 THERAPY STRATEGIES

**FIGURE 1:**
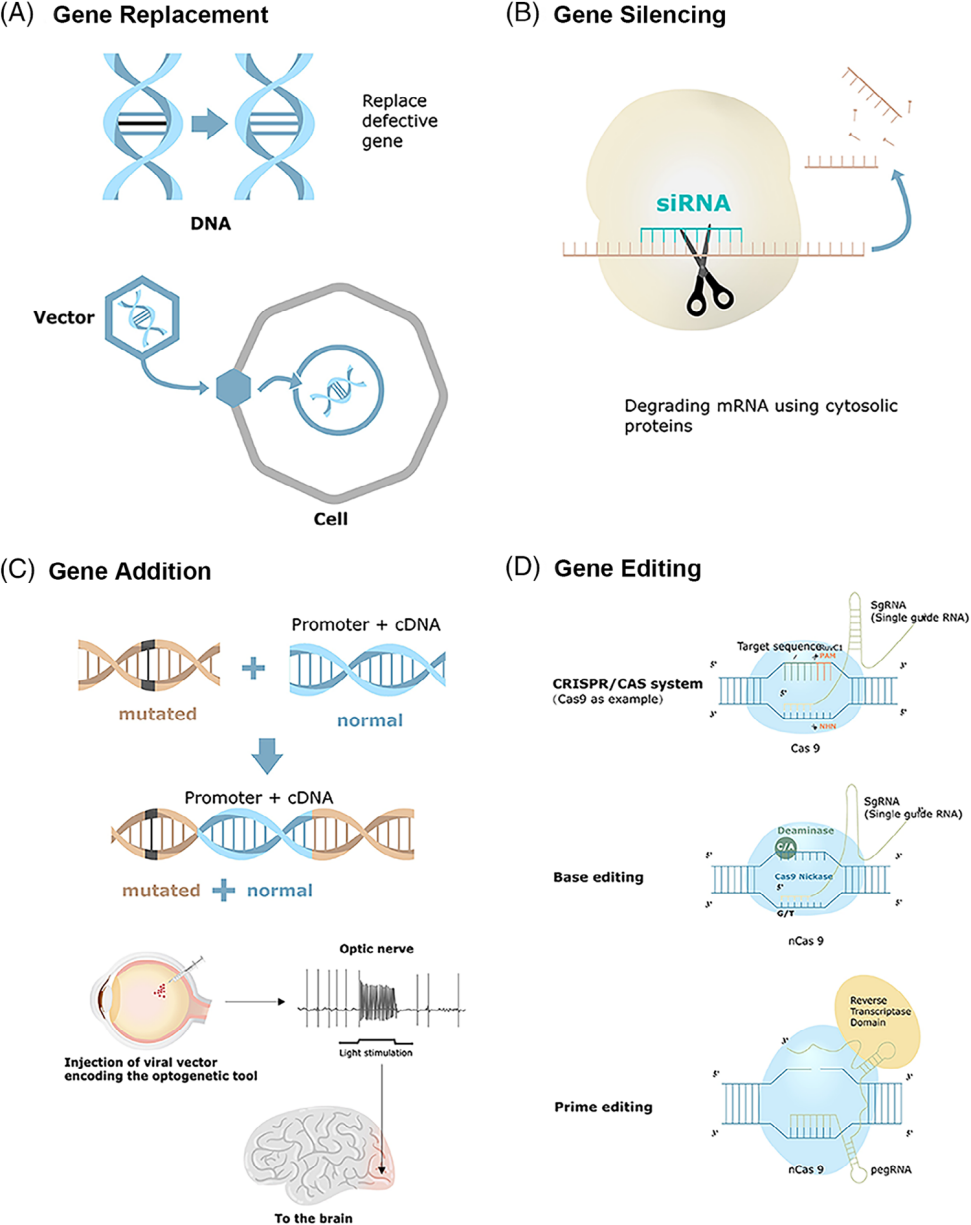
Different gene therapy strategies. Gene therapy includes gene replacement, silencing, addition, and editing. (A) Gene replacement entails delivering a gene product to compensate for loss‐of‐function mutations. (B) Gene silencing involves epigenetic modifications of genes, resulting in inactivation of previously active individual genes or larger chromosome regions. (C) Gene addition provides a functional gene copy into the cell or introduces another gene to potentially manage or treat a genetic disease. Optogenetics is a type of gene addition. (D) Gene editing is a strategy to make highly specific changes in DNA sequences. Gene editing mainly includes CRISPR–Cas system, base editing, and prime editing.

Gene therapy is modality for managing acquired or inherited diseases by introducing genetic information into target cells to correct or mitigate a disease caused by a gene defect or abnormal gene expression.^7^, ^8^ It involves therapeutic addition, repair, or alteration of defective genes by functional counterparts to restore or diminish gene expression activities^9^ (Figure 1). Its broader definition includes vaccine development, immunotherapy, particularly cancer immunotherapy, and oligonucleotide and RNA interference (RNAi)‐based gene silencing.^10^

### 2.1 Gene replacement

Gene replacement introduces a functional gene to compensate for deficiencies due to loss‐of‐function mutations, particularly effective in addressing recessive monogenic diseases. Adeno‐associated virus (AAV) is favored for gene therapy owing to its small size, lack of human pathogenicity, weakened postinjection immune responses, and engineering‐friendliness.^11^ However, this method cannot tackle all gene mutations, including dominant mutations, polygenic mutation conditions, and large genes incompatible with delivery vectors.^12^ Restoring protein synthesis at suboptimal physiological levels can alleviate symptoms in various circumstances. In certain diseases, transferring functional genes to a restricted set of cells in the impacted tissue can be sufficient to bring about a healing effect. Numerous current gene replacement clinical trials have shown promising treatment results, including trials for conditions like retinitis pigmentosa (RP)^13^ and choroideremia.^14^

### 2.2 Gene silencing

Gene silencing primarily addresses monogenic diseases due to gain‐of‐toxic mutations, with recombinant adeno‐associated viruses (rAAVs)‐based RNAi strategies currently dominating gene silencing platforms.^15^ CRISPR–Cas13a provides a flexible platform to reduce gene expression at the mRNA level in mammalian cells and therapeutic development.^16^ A dual AAV8 system can silence gene transcription.^17^ Ocular small interfering RNA (siRNA) gene‐silencing therapy centered on siRNA drugs is currently developed for treating neovascular age‐related macular degeneration (AMD) and glaucoma, with ongoing clinical investigation.^18^, ^19^ Preclinical trials of rAAV‐based gene silencing aimed at the vascular endothelial growth factor (VEGF)/phosphatidylinositol 3‐kinase (PI3K)/protein kinase B, PKB (Akt) pathway in diabetic retinopathy (DR) have shown promising results in decreasing the permeability of retinal blood vessels in DR rats.^20^ However, gene silencing therapies face several challenges, including RNA instability, poor bioavailability, toxicity, and off‐target effects. To date, no clinical trial of this strategy in the ocular field has advanced beyond phase III.^18^

### 2.3 Gene addition

Gene addition is a potential therapy strategy to tackle complex genetic diseases and acquired disorders.^21^ It can modulate diseases in a variety of ways by providing various factors for neurological disorders and altering signaling pathways.^22^, ^23^ This approach utilizes rAAV to deliver genes capable of producing recombinant antibodies that neutralize HIV1 infections, functioning similarly to an effective HIV‐1 vaccine.^24^ However, the efficacy of this strategy may be limited by immune responses triggered by rAAV vectors. Further investigation is needed for gene addition in ocular diseases.

### 2.4 Gene editing

Gene editing involves modifying a specific DNA into a different, preferred DNA or DNAs within the original genome structure. The process typically involves two stages: creating targeted DNA breaks in the genome and repairing these breaks, ultimately leading to the intended DNA modification. A range of programmable nucleases have been generated to induce these DNA breaks, including engineered Cas proteins, meganucleases (EMNs), zinc finger nucleases (ZFNs), and transcription activator‐like effector nucleases (TALENs).^25^ Among them, Cas proteins are extensively studied due to their ease of programming to target specific genomic DNA loci.^26^ Nonhomologous end joining (NHEJ) and homology‐directed repair (HDR) become the two fundamental cellular DNA repair processes that lead to therapeutic gene‐editing effects. NHEJ entails direct joining of the cleaved strands, leading to insertions or deletions, and is primarily employed for gene disruption. By contrast, HDR utilizes a guided amendment technique and an external repair template containing the proper nucleotide sequence to mediate the process.^27^

#### 2.4.1 DNA editing

CRISPR–Cas systems target particular DNA sequences with the assistance of guide RNA, which recognizes the protospacer adjacent motif (PAM).^28^ Of the two naturally existing CRISPR–Cas immune systems, the class 1 (types I, III, and IV) system applies multiprotein assemblies to split nucleic acids, whereas the class 2 systems (types II, V, and VI) deploys a single protein effector domain for cleavage.^29^ Notably, Cas9, Cas12, and Cas13 in the class 2 system are currently the subjects of investigation.^30^ For gene targeting and genome editing applications, class 2 CRISPR‐associated nucleases serve as adaptable tools for nucleic acid detection and handling.^31^

#### 2.4.2 Base editing

##### Adenine and cytosine base editors

Base editors are extensively utilized to explore and address genetic disorders across various cell types and animal models representing human genetic diseases.^32^, ^33^, ^34^, ^35^ Adenine base editors (A•T to G•C base editors; ABE) and cytosine base editors (C•G to T•A base editors; CBE) offer precise genome editing alternatives without inducing DSBs, thus avoiding the risk of genomic instability and unpredictable outcomes associated with DNA repair. ABEs are particularly useful for examining and rectifying disease‐causing alleles, as transforming an A•T pair into a G•C pair theoretically fixes almost 50% of harmful point mutations.^30^, ^36^ Recent progress has resulted in the creation of updated base editors with increased editing efficiency at challenging target sites, broader editing windows, and decreased the formation of undesirable products.^37^, ^38^, ^39^ Similarly, CBEs have evolved into new generations to overcome target sequence context constraints, offering higher editing efficiency at challenging target sites and broader editing windows.^39^ Dual‐AAV delivering split base editors have enabled effective in vivo base editing in recent research.^40^, ^41^, ^42^, ^43^ Continued refinement of Cas domains and deaminases holds the potential to facilitate single‐AAV editing in the future. Overall, the progression of base editing broadens the range and feasibility of genome editing, promising to enhance the variety and effectiveness of accurately implemented genome alterations. Base editing eliminates the dependence on random DNA repair mechanisms, donor templates, and dsDNA cleavage, which vary according to cell state and type, and improves editing efficiency in cells without the requirement for template‐based, HDR, yielding precise, predictable, and efficient genetic outcomes at specified sequences. Future precision medicine may concentrate on rectifying single‐point mutations.

##### RNA editing

Another category of base editing is RNA base editors, targeting A‐to‐I and C‐to‐U corrections. Although DNA base editing offers permanent, irreversible alterations to the genome, RNA base editing allows for reversible alterations to the cells’ genetic code or RNA's epi‐transcriptomic modifications.^36^ In certain clinical situations, site‐directed RNA editing may be a safer or more efficient option than genome editing, providing a natural editing equilibrium without off‐target editing and avoiding ectopic protein expression.^44^, ^45^ Since the identification of Cas13, many CRISPR‐based RNA base editing approaches have been developed. An engineered Cas13 variant can robustly knock down and edit RNA, offering a platform with broad applications in research and biotechnology, including modifying harmful mutations.^46^ RNA editing for specific C‐to‐U exchange systems (RESCUE) have the capability to edit endogenous transcripts and modulate posttranslational protein modification, such as phosphorylation.^47^ Abudayyeh et al.^47^ engineered a novel C‐to‐U RNA editor capable of directing A‐to‐I adenosine deaminase to act on transcripts in mammalian cells. This method signifies substantial progress in RNA base editing, paving the way for developing more diverse types of RNA base editors.^47^

#### 2.4.3 Prime editing

Prime editing (PE) represents an adaptable and accurate genome modification method, enabling the direct incorporation of new genetic material at a targeted DNA locus. This technique employs a catalytically hindered Cas9 endonuclease combined with a custom‐designed reverse transcriptase guided by a PE guide RNA (pegRNA) to achieve its editing objectives.^48^ PE works in various cells, organoids, and mice embryos with varying degrees of success.^48^, ^49^, ^50^ PE2 rescued the expression of an mRNA/long noncoding RNA gene pair by precisely editing a single base at a transcription factor binding site, thus broadening the genome editing toolkit.^51^ Chen et al.^52^ developed PE4 (PE2 plus a dominant negative mismatch repair protein), PE5 (PE3 plus a dominant negative mismatch repair protein), and PEmas (synergy with PE4, PE5, and engineered pegRNAs) systems, greatly enhancing PE efficiency and outcome purity at numerous inherent genomic locations in mammalian cells. Recently, split PE has become increasingly popular. Zheng et al.^53^ successfully delivered a dual‐AAV8 split prime editor into mice. Liu et al.^54^ improved the method by splitting pegRNAs into a single guide RNA (sgRNA) and a circular RNA RT template, thereby boosting its adaptability and stability.

PE has manifested superior or equivalent efficiency to HDR while producing fewer byproducts. It presents a distinct array of advantages and drawbacks compared with base editing. Moreover, it incurs substantially fewer off‐target modifications than Cas9 at identified Cas9 off‐target positions^48^ and provides more targeting versatility.^30^, ^55^ PE also enables the effective generation of tiny deletions and insertions, expanding its applicability to almost 90% of disease‐associated mutations.^56^

Gene editing techniques provide accurate tools for gene insertion, deletion, and rectification.^57^ CRISPR–Cas genome editing systems have revolutionized life science research and driven medicine translation.^58^ The advancement of gene therapy has relied on multiple pivotal factors, such as the refinement of gene delivery methods, comprehension of the genetic origins of diseases, creation of novel nucleic acid‐based therapies, and innovation in genome engineering technologies.^59^ Improvements in the stability, safety, and pharmacokinetic profiles of gene delivery vectors are instrumental in evoluting gene therapy.^59^

## 3 THERAPY DELIVERY SYSTEMS

### 3.1 Viral delivery system

**FIGURE 2:**
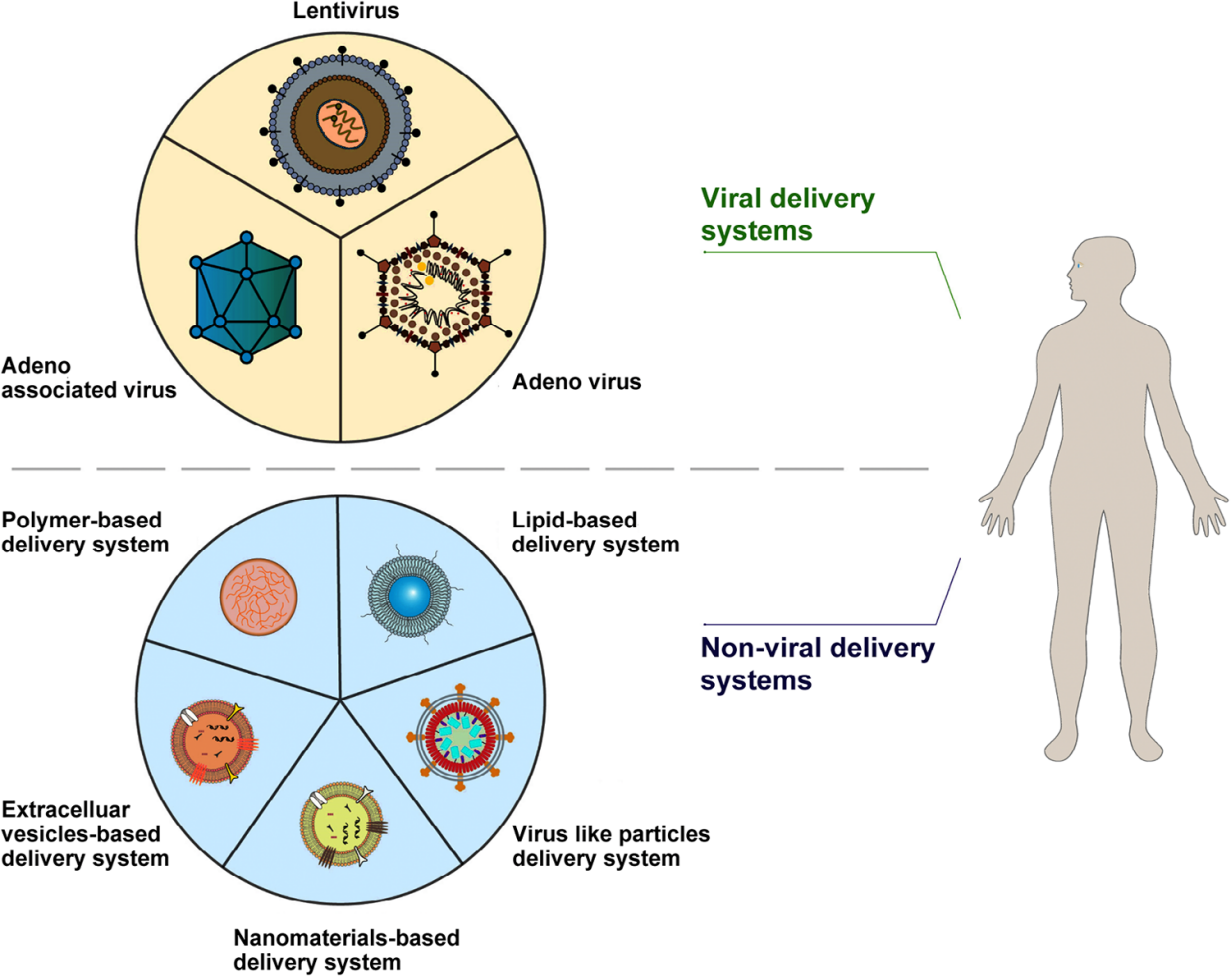
Gene therapy delivery systems are categorized as viral system and nonviral system. Viral systems include lentiviruses, adenoviruses, and adeno‐associated viruses. Nonviral systems mainly include polymer‐based, lipid‐based, virus‐like particle‐based, nanomaterials‐based, and extracellular vesicles‐based delivery systems.

Gene delivery faces many challenges, including determining the optimal delivery method, addressing considerations such as gene size, vector immunogenicity and specificity, and production issues such as cost and process, all of which require rigorous academic investigations.^12^ Viral vectors stand out as the preferred carriers for delivering DNA due to their exceptional efficiency.^60^ Although gene therapy predominantly relies on viral vectors for gene delivery, nonviral vectors have surfaced as complementary approaches to overcome certain drawbacks of viral vectors, including immunogenicity and packaging constraints.^61^ A diverse array of viral and nonviral vectors provide numerous possibilities (Figure 2). Various gene delivery strategies are discussed in the following sections.

#### 3.1.1 Retroviruses and lentiviruses

Retroviruses and lentiviruses, both enveloped single‐stranded RNA viruses,^62^ share a similar genomic structure. Their genomes contain the env, gag, and pol genes flanked by long terminal repeats, which harbor enhancer/promoter elements necessary for integrating the host cells’ genome via double‐stranded DNA (dsDNA) provirus formation.^63^ Retroviruses can package approximately 8 kb of exogenous sequences.^63^ They execute reverse transcription, converting their RNA genome into dsDNA replicas. These DNA copies can subsequently be integrated into the host genome.^64^ However, retrovirus vectors can only transduce dividing cells, posing a significant hurdle for gene therapy applications.^65^ In contrast, lentivirus vectors are capable to transduce dividing and nondividing mammalian cells,^66^ making them an attractive tool for permanent genome modification.^67^ Clinical trials have underscored the therapeutic effectiveness of lentiviral vectors in various diseases, including ocular disorders. Lentivirus vectors have shown their capacity to transduce various ocular tissues following intravitreal injection, including retinal pigment epithelium (RPE), trabecular meshwork, corneal endothelium, ciliary body, anterior chamber, iris pigmented epithelium, inner nuclear layer, and ocular muscles,^68^ with subretinal injection being safe, tolerable, with no dose‐constraining toxicities, mild ocular inflammation, and reproducible, prolonged transgene expression.^69^ In ocular gene therapy, both retroviruses and lentiviruses have been utilized in the development of RetinoStat for treating neovascular AMD (ClinicalTrials.gov Identifier: NCT01301443 and NCT01678872). Continuous monitoring of individuals undergoing lentiviral vector‐mediated gene therapies remains essential to assess long‐term safety and efficacy. Despite progress, further basic and clinical research is still required to enhance production and transduction efficiency.

#### 3.1.2 Adenoviruses

Adenoviruses are nonenveloped, linear, dsDNA viruses with genomic sizes typically around 40 kb.^70^ Their wide range of host cell tropisms enables them to infect host cells regardless of their cell division status.^71^ Unlike retroviruses, adenovirus infection is independent of the cell‐cycle phase and does not involve integrating viral genes into the host genome.^72^ Human adenoviruses encompass over 100 subtypes (serotypes 1−52 and genotypes 53−103).^72^ Engineered adenovirus vectors have found applications in gene therapy, cancer therapy, and vaccine development.^73^, ^74^, ^75^ Clinical trials of adenoviral gene therapy have revealed mild inflammatory response, the capability to infect different cells, and a low risk of chromosome mutagenesis, offering considerable safety and effectiveness in in vivo gene therapy.^76^, ^77^ Adenoviral vectors are extensively utilized in gene therapy research, with notable progress observed in in vivo and ex vivo genome editing, independently or in combination with other vectors, despite facing certain challenges.^78^, ^79^

#### 3.1.3 Adeno‐associated viruses

**FIGURE 3:**
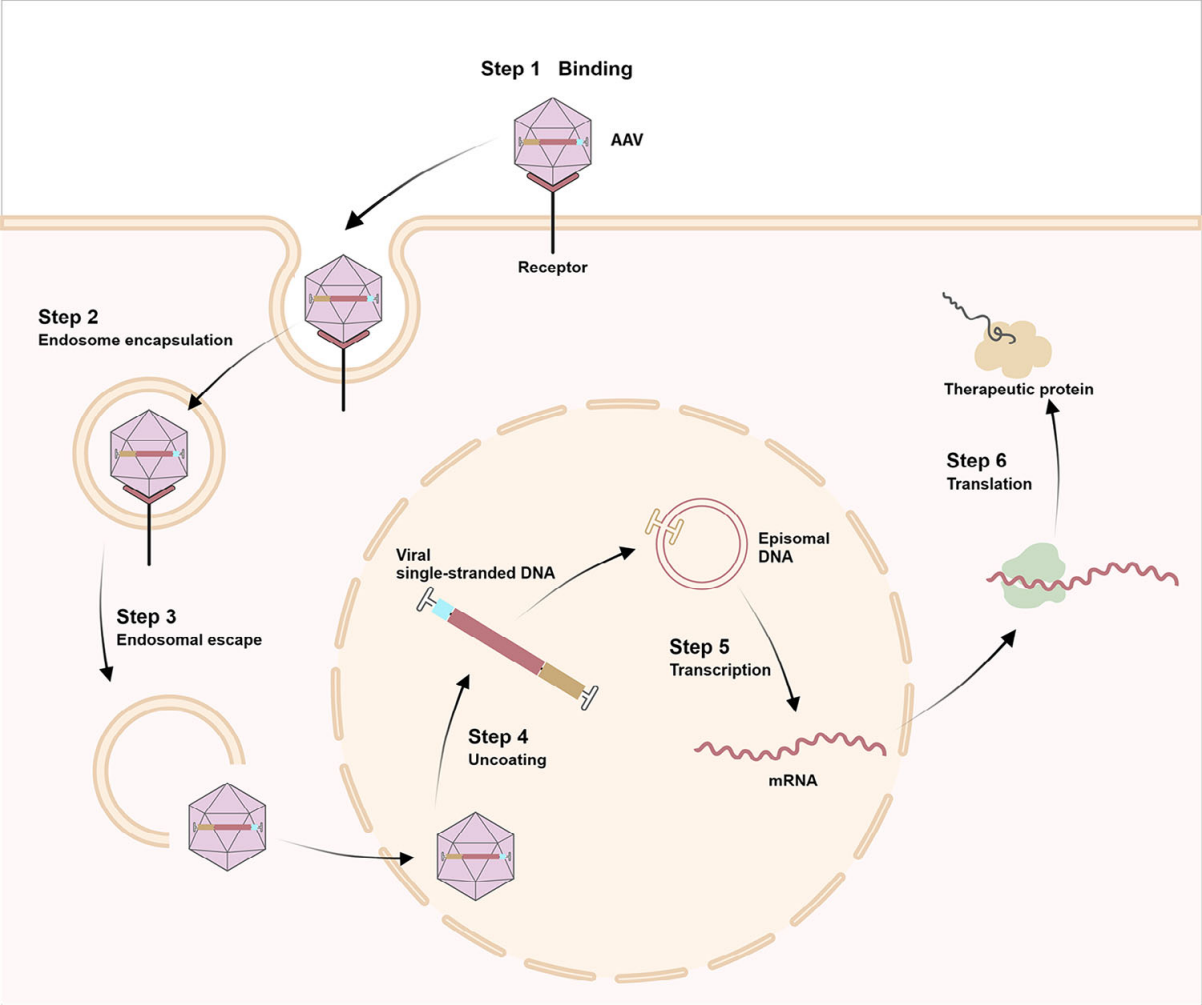
AAV transduction steps in target cells. Step 1: AAV vectors bind to the cell surface receptor. Step 2: AAV vectors are internalized into endosomes via endocytosis. Step 3: Undegraded AAV virions are intracellularly transported to the nucleus after release from endosomes. Step 4: In the nucleus, AAV genome is released from AAV virions and subsequently transcribed to dsDNA. Step 5: After transcription, mRNA is exported to the nucleus for translation. Step 6: mRNA is translated into therapeutic proteins.

AAV is a small nonenveloped, single‐stranded DNA (ssDNA) genome encapsulated within an icosahedral capsid, about 22−26 nm in diameter.^80^ It comprises a 4.7 kb ssDNA genome flanked by inverted terminal repeats (ITRs). These ITRs, spanning 145 nucleotides, can self‐anneal into hairpin forms and are essential for genome encapsulation following replication and function as packing signals.^81^ AAV1–AAV13 represent 13 distinct serotypes found in human and nonhuman primates.^82^ AAV genome is characterized by its highly condensed structure, incorporating coinciding coding regions, alternative splicing patterns, and multiple translation initiation sites derived from canonical and noncanonical start codons.^80^ rAAVs are the most prevailing gene delivery vectors,^83^ produced by replacing viral sequence with a transgenic cassette.^84^ The capsid, genome, and transgene product are AAV vectors’ primary immunogenic components,^85^ with intravitreal administration inducing dose‐dependent inflammatory responses.^85^ Despite this, AAVs exhibit lower immunogenicity than other viral vectors.^86^ AAV‐based gene therapy has exhibited safety and sustained effectiveness in several preclinical trials.^83^, ^85^ Figure 3 illustrates AAV vectors’ transduction mechanism.

### 3.2 Nonviral delivery system

Nonviral carriers have emerged as up‐and‐coming alternatives to viral vectors due to their advantageous safety attributes.^87^, ^88^ Hybrid molecules combining polycationic and polyanionic polymers with other organic and inorganic substances like lipids, magnetite, or polymers can efficiently transfer genetic material for various applications.^89^, ^90^ Polyplexes, composed of cationic biodegradable polymeric materials, offer potential as alternatives to viral vectors due to their biocompatibility and self‐assembly capabilities.^91^ Guo et al.^92^ developed a novel gene carrier, zwitterionic polyplexes, through a two‐step process to boost gene transfection efficiency in vitro and in vivo. This innovative tactic demonstrates superior efficacy in gene delivery.^92^ Another nonviral delivery method utilizing a highly branched poly (β‐amino ester) polymer has also been developed to facilitate genomic editing via CRISPR/Cas9‐mediated targeted excision of exon 80 in the COL7A1 gene. This innovative strategy incorporates a dual‐guide RNA sequence system for precise and efficient gene modification.^93^ Li et al.^94^ presented a genome‐editing strategy utilizing CRISPR/Cas9 technology, employing a meticulously designed semiconducting polymer brush. Through laser stimulation, the nanocomplexes formed by the semiconducting polymer brush and CRISPR/Cas9 cassette demonstrate remarkable efficacy in achieving site‐specific and accurate genome editing in vitro and in vivo while exhibiting low toxicity.^94^ Another important nonviral delivery approach is lipidic delivery systems. Lipid nanoparticles have been utilized to formulate Patisiran^®^ to intravenously administer siRNA into the liver to reduce transthyretin production.^95^ The field of nonviral CRISPR/Cas cargo delivery is progressing, with ongoing clinical trials exploring its potential for various therapeutic applications.^96^ CRISPR ribonucleoprotein (RNP)‐based genetic engineering has emerged as an appealing method with numerous benefits. The swift degradation of RNPs allows for precise dosage titration while maintaining high editing efficiency, enabling the editing procedure to be conducted without the need for DNA or transgenes, resulting in minimal off‐target effects.^97^ Prior investigations into the nonviral administration of nanoparticles to the retina have exhibited promising but varying degrees of success. Notably, Kim et al.^98^ showcased the genome editing efficacy of subretinal infusing SpCas9 with a gRNA specifically targeting the vascular endothelial growth factor (VEGF) gene in retinal pigment epithelial cells in vivo. Banskota et al.^99^ formulated engineered DNA‐free virus‐like particles (eVLPs) that effectively packaged and delivered base editor or Cas9 to improve overall AAV or plasmid delivery efficiency. Employing various glycoproteins in eVLPs modified their cellular tropism and partly restored visual function in a mouse genetic blindness model.^99^ RNP‐mediated base editing offers advantages over plasmid or viral vector‐based gene editing, exhibiting reduced off‐target effects. Jang et al.^100^ purified CBE/ABE proteins from human cells and utilized NG PAM‐targetable ABE RNPs for in vivo gene modification in rd12 model mice, demonstrating decreased off‐targets in both DNA and RNA compared with plasmid‐encoded ABE. With their excellent biocompatibility, prolonged circulation, and genetic engineering capabilities, exosomes have gained prominence as nonviral gene delivery vectors. As natural carriers for intercellular communication, they can encapsulate various biological payloads, such as miRNAs and siRNAs, offering versatile options for therapeutic applications.^101^ It may be feasible to regulate mutant gene expression spatiotemporally without the need for integration into the host genome using nonviral systems. Considering various cell types in the retina, cell‐specific regulation via a promoter or ligand may enhance the specificity and effectiveness of nonviral gene therapy. Although nonviral vectors possess advantages of nontoxicity, low immune response, and mass production, their effectiveness remains a challenge. Before the current strategy can be used on humans, some issues must be resolved in preclinical studies. Despite these drawbacks, the future of nonviral gene delivery is promising.

Despite encouraging outcomes, numerous obstacles must be addressed before gene therapy can be applied in clinical cases, with safety concerns being the most important. The advent of novel techniques for gene transfer into cells or genome manipulation calls for extreme caution and a thorough analysis of all potential effects before approval. The development of vectors has prompted research into host‐vector interactions. The utilization of viral gene therapy vectors leverages viruses’ inherent capacity to administrate genetic material to the host cells’ nuclei for endogenous gene expression. A complete comprehension of viral biology and genome organization will propel virus genome engineering. Moreover, an in‐depth comprehension of the molecular interactions occurring during transduction would help develop and optimize these vectors, paving the way for promising gene transfer vectors.

## 4 CURRENT AAV GENE THERAPY STRATEGIES IN HUMAN DISEASES

**FIGURE 4:**
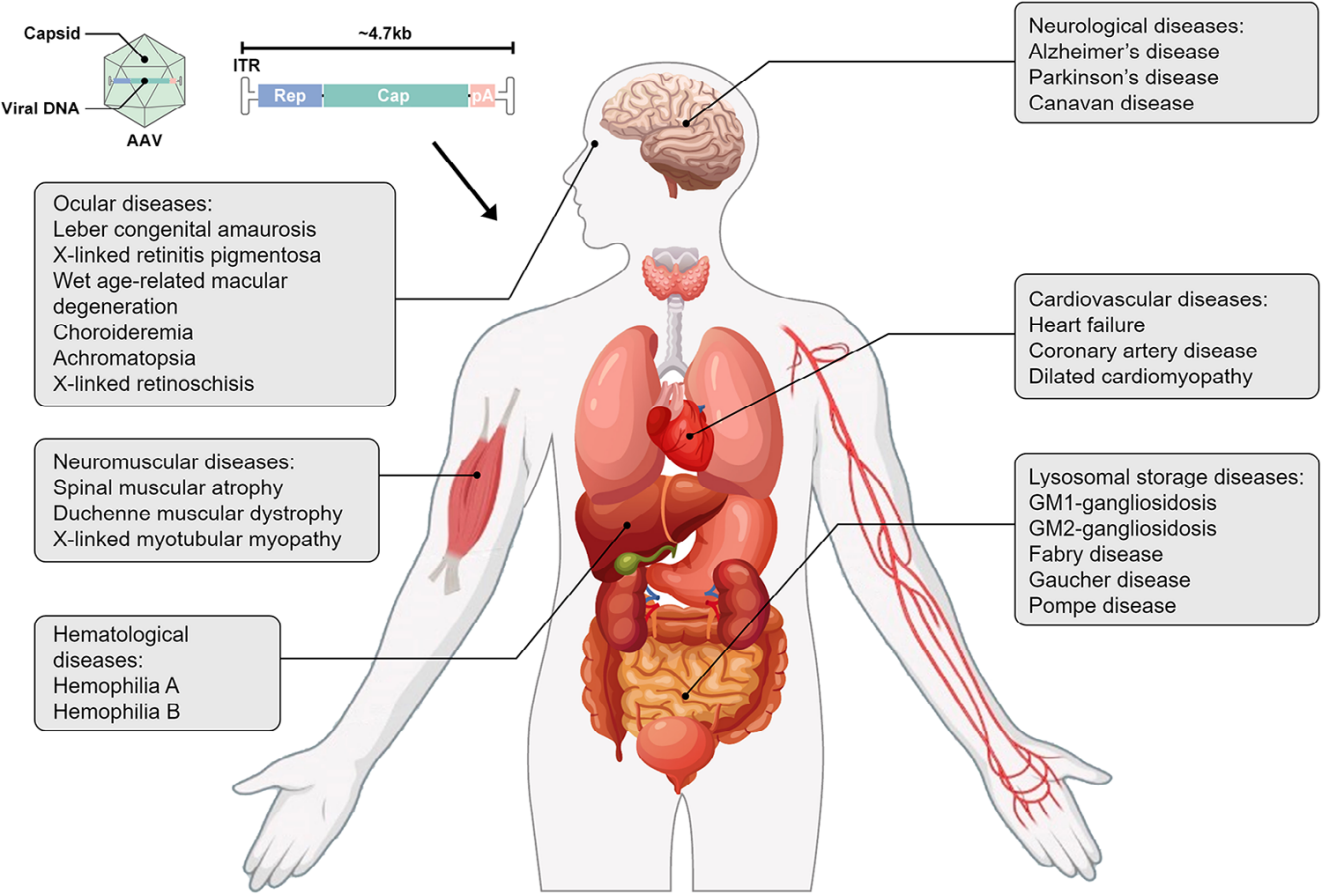
The outline of AAV‐mediated gene therapy in human diseases. AAV‐mediated gene therapy in human diseases covers a wide range of conditions, including ocular, neuromuscular, hematological, neurological, cardiovascular, and lysosomal storage diseases.

**FIGURE 5:**
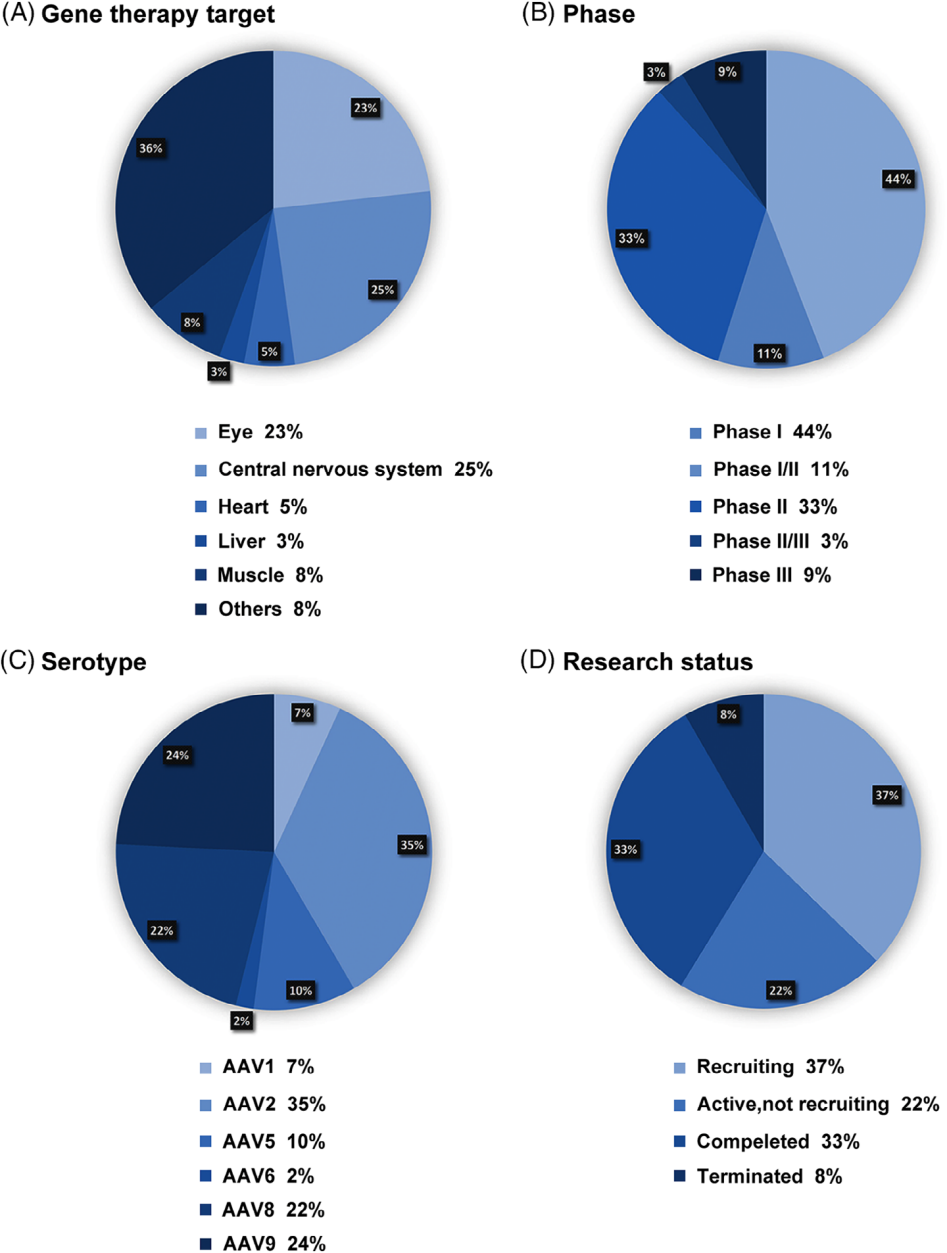
Summary of AAV‐mediated gene therapy clinical trials. 387 clinical trials (ClinicalTrials.gov) related to AAV‐mediated gene therapy in humans are listed. The registered trials are categorized based on gene therapy target (A), phase (B), serotype (C), and research status (D).

Gene therapy has seen remarkable advancements in recent decades, demonstrating its immense potential in treating a broad spectrum of genetic and acquired diseases. Several AAV gene therapy products have been introduced, targeting diverse conditions. This section explores the clinical applications of AAV across significant human diseases, including ocular, neuromuscular, hematological, neurological, cardiovascular, and lysosomal storage diseases (LSDs) (Figure 4). Figure 5 provides a concise overview of 387 clinical trials. Tables 1 and 2 show the properties and clinical utilization of various AAV serotypes.

**TABLE 1:** Features and clinical use of AAV serotypes (*Data sources*—/www.clinicaltrials.gov/).

AAV serotypes	Origin of isolation	Primary receptor	Coreceptors	Tropism	ClinicalTrials.gov identifier
AAV1	Monkey	Sialic acid	AAVR	CNS, Muscle, heart	Limb girdle muscular dystrophy type 2C (NCT01344798), frontotemporal dementia and progranulin mutations (NCT04747431), chronic heart failure (NCT00534703)
AAV2	Human	HSPG	FGFR1, HGFR, αVβ5 integrin, LR, and CD9	Liver, CNS, heart, retina	Mild cognitive impairment and Alzheimer's disease (NCT05040217), LCA (NCT00999609)
AAV3	Human	HSPG	FGFR1, LR, and HGFR	Muscle, liver, heart, retina	No trials underway
AAV4	Monkey	Sialic acid	/	CNS, retina	LCA (NCT01496040)
AAV5	Human	Sialic acid	PDGFR	CNS, lung, retina	Huntington's disease (NCT05243017), coronavirus infection (NCT05037188), XLRP (NCT05926583)
AAV6	Human	Sialic acid and HSPG	AAVR, and EGFR	CNS, lung, muscle, heart	CNS diseases (NCT02702115)
AAV7	Monkey	/	/	CNS, liver, muscle	No trials underway
AAV8	Monkey	LR	/	Liver, muscle, pancreas, CNS, retina	XLRP (NCT03116113), DMD (NCT05683379)
AAV9	Human	Terminal N‐linked galactose	Putative integrin, LR	CNS, lung, liver, heart, muscle	Canavan disease (NCT04998396), spinal muscular atrophy (NCT03505099)
AAV10	Monkey	/	/	Muscle	No trials underway
AAV11	Monkey	/	/	/	No trials underway
AAV12	Human	Mannosamine and mannose have been recommended as components of a potential receptor complex	Nasal, muscles	No trials underway	
AAV13	/	/	HSPG	/	/

Abbreviations: AAV, adeno‐associated viruses; AAVR, AAV receptor; CNS, central nervous system; HSPG, heparan sulfate proteoglycan; FGFR1, fibroblast growth factor receptor 1; HFGR, hepatocyte growth factor receptor; LR, laminin receptor; PDGFR, platelet‐derived growth factor receptor; EGFR, epidermal growth factor receptors; LCA, Leber congenital amaurosis; XLRP, X‐linked retinitis pigmentosa; DMD, Duchenne muscular dystrophy; CD9, a member of the transmembrane 4 superfamily also known as the tetraspanin family.

**TABLE 2:** A selection of ongoing rAAV interventional clinical trials (*Data sources*—Https://www.clinicaltrials.gov/).

Gene therapy target	Disease	Intervention	Delivery	Strategy	Phase	ClinicalTrials. gov identifier
Eye	Achromatopsia	rAAV2tYF‐PR1.7‐hCNGB3	rAAV2	Replacement	Phase 1+2	NCT02599922
Eye	Achromatopsia	AAV2/8‐hCARp.hCNGB3	AAV2/8	Replacement	Phase 1+2	NCT03001310
					Completed	
					Has results	
Eye	Achromatopsia	AGTC‐402	rAAV2	Replacement	Phase 1+2	NCT02935517
Eye	Achromatopsia	AAV2/8‐hCARp.hCNGB3 and AAV2/8‐hG1.7p.coCNGA3	AAV2/8	Replacement	Phase 1+2	NCT03278873
Eye	Achromatopsia	AAV2/8‐hG1.7p.coCNGA3	AAV2/8	Replacement	Phase 1+2	NCT03758404
					Completed	
					Has Results	
Eye	Choroideremia	rAAV2.REP1	rAAV2	Replacement	Phase 1+2	NCT01461213
Eye	Choroideremia	AAV2‐hCHM	AAV2	Replacement	Phase 1+2	NCT02341807
Eye	Choroideremia	rAAV2.REP1	AAV2	Replacement	Phase 1+2	NCT02671539
Eye	Choroideremia	BIIB111(AAV2‐REP1)	AAV2	Replacement	Phase 3	NCT03584165
Eye	Choroideremia	4D‐110(AAV vector carry human CHM gene)	AAV	Replacement	Phase 1	NCT04483440
Eye	LCA	AAV2‐hRPE65v2	AAV2	Replacement	Phase 1	NCT00516477
Eye	LCA	AAV2‐hRPE65v2	AAV2	Replacement	Phase 3	NCT00999609
Eye	LCA	AAV2/5‐OPTIRPE65	AAV2/5	Replacement	Phase 1+2	NCT02781480
Eye	LCA	rAAV2‐CB‐hRPE65	rAAV2	Replacement	Phase1+2	NCT00749957
Eye	LCA10	EDIT‐101	AAV	Replacement	Phase1+2	NCT03872479
Eye	LCA5	AAV8.hLCA5	AAV8	Replacement	Phase1+2	NCT05616793
Eye	LHON	scAAV2‐P1ND4v2	scAAV2	Replacement	Phase 1	NCT02161380
Eye	LHON	GS010(rAAV2/2‐ND4)	rAAV2/2	Replacement	Phase3 (REVERSE)	NCT02652780
Eye	LHON	NR082	AAV2	Replacement	Phase 1+2	NCT05293626
Eye	LHON	NFS‐02	AAV2	Replacement	Phase 1+2	NCT05820152
Eye	RP	AAV2/5‐hPDE6B	AAV2/5	Replacement	Phase 1+2	NCT03328130
Eye	RP	rAAV.hPDE6A	rAAV	Replacement	Phase 1+2	NCT04611503
Eye	XLRP	BIIB112(AAV8‐coRPGR)	AAV8	Replacement	Phase 1+2	NCT03116113
Eye	XLRP	AAV2/5‐RPGR	AAV2/5	Replacement	Phase 1+2	NCT03252847
Eye	XLRP	AAV5‐hRKp.RPGR	AAV5	Replacement	Phase 3	NCT05926583
Eye	X‐linked retinoschisis	AAV‐RS1(AAV8‐scRS/IRBPhRS)	AAV8	Replacement	Phase 1+2	NCT02317887
Eye	X‐linked Retinoschisis	rAAV‐hRS1	rAAV2	Replacement	Phase 1+2	NCT02416622
Eye	X‐linked Retinoschisis	ATSN‐201 (AAV.SPR‐hGRK1‐hRS1syn)	AAV	Replacement	Phase 1+2	NCT05878860
Eye	Neovascular AMD	RGX‐314(AAV vector containing a coding sequence for an anti‐VEGF protein)	rAAV	Silencing	Phase 1+2	NCT03066258
Eye	Neovascular AMD	ADVM‐022	AAV.7m8‐aflibercept	Silencing	Phase 1	NCT03748784
Eye	Neovascular AMD	KH631(AAV vector containing a coding sequence for an anti‐VEGF protein)	AAV	Silencing	Phase 1	NCT05657301
Eye	BCD	ZVS101e(rAAV8‐hCYP4V2)	rAAV8	Replacement	Phase 1+2	NCT05832684
Eye	BCD	VGR‐R01(CYP4v2‐coding gene delivered by AAV vector)	AAV	Replacement	Phase 1	NCT05694598
CNS	Alzheimer's disease	AAV‐hTERT	AAV	Addition	Phase 1	NCT04133454
CNS	Alzheimer's disease	AAV2‐BDNF(Brain‐Derived Neurotrophic Factor)	AAV2	Addition	Phase 1	NCT05040217
CNS	Alzheimer's disease	AVB‐101(AAV9 vector carry GRN gene)	AAV9	Addition	Phase 1+2	NCT06064890
CNS	Alzheimer's disease	PBFT02(AAV1 carry GRN gene)	AAV1	Addition	Phase 1+2	NCT04747431
CNS	Parkinson's disease	AAV2‐GDNF	AAV2	Addition	Phase 1	NCT04167540
CNS	Parkinson's disease	VY‐AADC01(AAV2‐hAADC)	AAV2	Addition	Phase 1	NCT01973543
CNS	Parkinson's disease	LY3884961	AAV9	Addition	Phase 1+2	NCT04127578
CNS	Canavan disease	AAV9 BBP‐812	AAV9	Replacement	Phase 1+2	NCT04998396
CNS	Canavan disease	rAAV‐Olig001‐ASPA	rAAV	Replacement	Phase 1+2	NCT04833907
CNS	Aromatic l‐amino acid decarboxylase deficiency	AAV2‐hAADC	AAV2	Replacement	Phase 2	NCT02926066
CNS	Aromatic l‐amino acid decarboxylase deficiency	VGN‐R09b(AAV9 carry human AADC gene)	AAV9	Replacement	Early Phase 1	NCT05765981
Heart	Heart failure	AAV1/SERCA2a	AAV1	Addition	Phase 2	NCT00534703
Heart	Heart failure	AAV9‐cTnT‐modTERT	AAV9	Addition	Early Phase 1	NCT05837143
Liver	Hemophilia A	Recombinant AAV2/6 human factor VIII gene therapy	rAAV2/6	Replacement	Phase 3	NCT04370054
Liver	Hemophilia A	BAY2599023(AAV hu37‐mediated gene transfer of B‐domain deleted human factor VIII)	AAV	Replacement	Phase 1+2	NCT03588299
Liver	Hemophilia A	AAV vector‐mediated gene transfer of human factor VIII	AAV5	Replacement	Phase 1+2	NCT03520712
Liver	Hemophilia A	AAV8 vector expressing B‐domain deleted factor VIII	AAV8	Replacement	Phase 1+2	NCT03370172
Liver	Hemophilia B	AAV5‐hFIX	AAV5	Replacement	Phase 1+2	NCT02396342
Liver	Hemophilia B	AAV5‐hFIXco‐Padua	AAV5	Replacement	Phase 3	NCT06003387
Liver	Hemophilia B	AAV8 Vector Expressing FIX Padua	AAV8	Replacement	Phase 1+2	NCT04394286
Liver	MPS‐I	SB‐318 [zinc finger nucleases (ZFN1, ZFN2, and hIDUA donor)	rAAV2/6	Editing	Phase 1+2	NCT02702115
Liver	MPS‐II	SB‐913 [zinc finger nucleases (ZFN1, ZFN2, and hIDUA donor)]	rAAV2/6	Editing	Phase 1+2	NCT03041324
Liver	MPS‐IIIA	LYS‐SAF302(AAV serotype rh.10 carrying human N‐sulfoglucosamine sulfohydrolase)	AAV rh.10	Replacement	Phase 2+3	NCT03612869
Liver	MPS‐IIIA	scAAV9.U1a.hSGSH	AAV9	Replacement	Phase 1+2	NCT04088734
Muscle	Spinal muscular atrophy	AVXS‐101(AAV9 carrying the SMN gene)	AAV9	Replacement	Phase 1	NCT02122952
Muscle	Spinal muscular atrophy	EXG001‐307(AAV9 containing cDNA encoding the human SMN protein)	AAV9	Replacement	Phase 1+2	NCT05614531
Muscle	Spinal muscular atrophy	GC101(AAV9 carrying a codon‐optimized SMN coding sequence)	AAV9	Replacement	Phase 1+2	NCT05824169
Muscle	Duchenne muscular dystrophy	scAAV9.U7.ACCA	rAAV9	Replacement	Phase 1+2	NCT04240314
Muscle	Duchenne muscular dystrophy	rAAV2.5‐CMV‐minidystrophin	rAAV2.5	Replacement	Phase 1	NCT00428935
Muscle	Duchenne muscular dystrophy	rAAV1.CMV.huFollistin344	rAAV1	Replacement	Phase 1+2	NCT02354781
Muscle	X‐linked myotubular myopathy	AT132(AAV8 vector containing a functional copy of the human MTM1 gene)	AAV8	Replacement	Phase 2+3	NCT03199469
Muscle	Alpha 1‐antitrypsin deficiency	rAAV1‐CB‐hAAT	rAAV1	Replacement	Phase 1	NCT00430768
Muscle	Charcot–Marie–Tooth neuropathy type 1A	scAAV1.tMCK.NTF3	rAAV1	Addition	Phase 1+2	NCT03520751
Muscle	Dysferlinopathy	rAAVrh74.MHCK7.DYSF.DV	rAAVrh74	Replacement	Phase 1	NCT02710500

Abbreviations: AADC, aromatic l‐amino acid decarboxylase; AAVR, AAV receptor; BCD, Bietti's crystalline dystrophy; BDNF, brain‐derived neurotrophic factor; DMD, Duchenne muscular dystrophy; FGFR1, fibroblast growth factor receptor; HGFR, hepatocyte growth factor receptor; HSPG, heparan sulfate proteoglycan; LCA, Leber congenital amaurosis; LHON, Leber's hereditary optic neuropathy; LR, laminin receptor; MPS, mucopolysaccharidoses; neovascular AMD, neovascular age‐related macular degeneration; PDGFR, platelet‐derived growth factor receptor; rAAV; recombinant adeno‐associated viruse; RP, retinitis pigmentosa; SMA, spinal muscular atrophy; VEGF, vascular endothelial growth factor; XLMTM, X‐linked myotubular myopathy; XLR, X‐linked retinoschisis; XLRP, X‐linked retinitis pigmentosa; ZFN, zinc finger nucleases.

### 4.1 Ocular diseases

#### 4.1.1 Retinitis pigmentosa

RP, or rod‐cone dystrophy, represents a heterogeneous group of hereditary diseases featured by the initial loss of rod photoreceptor cells, succeeded by cone photoreceptors and RPE degeneration, eventually causing a gradual vision loss.^102^ The mechanism underlying RP involves a shortened rhodopsin due to mutations, which impair the cells’ capacity to fold and transport proteins, causing cell death via unfolded protein responses.^103^ Clinical manifestations of RP include nyctalopia, restricted visual fields, and eventually central vision loss.^104^, ^105^ Genes such as Usher syndrome type IIA (USH2A, Usherin), retinitis pigmentosa 2 homolog (RP2), and phosphodiesterase 6B (PDE6B) harbor harmful mutations associated with RP.^106^

RP is categorized into nonsyndromic and syndromic forms. The former exclusively causes retinal dystrophy without affecting other organs, with a prevalence of one in 5000 and is inherited as an autosomal‐dominant (about 30−40% of cases), autosomal‐recessive (50−60%), or X‐linked (5−15%) traits.^107^ The latter presents additional symptoms resulting from systemic disorders coexisting with retinal degeneration. Syndromic RP includes LCA, Usher syndrome, and Bardet–Biedl syndrome. LCA, with a prevalence of one in 80,000, is a RPE65 mutations‐caused autosomal recessive hereditary disease featured by early vision loss, congenital pupillary reaction defects, and nystagmus noticed during infancy.^108^, ^109^ Usher syndrome, with a prevalence of about one in 10,000, presents as classic RP clinical symptoms along with varying degrees of hearing loss and vestibular dysfunction from USH gene mutations.^110^

Luxturna (voretigene neparvovec) represents a pioneering gene therapy with AAV2 vectors carrying a modified form of the human RPE65 gene, designed specifically for individuals with biallelic disease‐causing variants in RPE65. In a phase III clinical trial involving 31 participants diagnosed with inherited retinal disease associated with biallelic RPE65 mutations, Luxturna administration yielded substantial improvements in visual function, with sustained benefits observed for up to 3–4 years. Notably, no severe adverse reactions were observed after 1 year (ClinicalTrials.gov Identifier: NCT00999609).^4^, ^5^, ^111^ Luxturna's success has spurred further investigation into gene abnormalities associated with hereditary retinal disorders, with numerous clinical trials ongoing and registered on clinicaltrials.gov. Technologies on efficient vector delivery, CRISPR/Cas9, and iPSCs‐based cell transplants are accelerating personalized precision RP treatment.^112^ CRISPR/Cas gene editing and gene regulation hold promise in improving the safety and efficacy of currently developed techniques, such as RNA editors. Although Luxturna exemplifies the potential of gene therapy, challenges remain in ensuring long‐term safety, developing efficient delivery systems, managing immune responses, and addressing the high costs associated with gene therapy.

#### 4.1.2 X‐linked RP

RP GTPase regulator (RPGR) gene mutations commonly underlie the inherited X‐linked retinal degeneration.^113^ Using sgRNA (Cas9) and AAV vectors, RPGR gene‐editing has shown promise in preserving photoreceptors in mouse models with RPGR‐related X‐linked RP (XLRP).^114^ Subretinal injection of CRISPR–cas9 AAV vectors has successfully reinstated the open reading frame of RPGR^ORF15^ in a subset of cells distributed widely in the retina of rd9 mice.^115^ A phase I/II clinical trial employing a dose‐escalation design, a single sub‐retinal injection of BIIB112, an AAV vector encoding codon‐optimized human RPGR (AAV8‐coRPGR), was conducted on individuals with XLRP resulting from RPGR mutation (ClinicalTrials.gov Identifier: NCT03116113), showing safety concerns primarily limited to steroid‐responsive subretinal inflammation in high dose groups. Encouragingly, six patients exhibited improvements in the visual field, commencing at 1 month and sustained until the last follow‐up assessment.^116^ A phase I/II dose escalation trial of rAAV2–RPGR for children and adults with XLRP due to faulty RPGR has been completed, with results pending publication (ClinicalTrials.gov Identifier: NCT03252847). Several other clinical studies are underway, including a phase I/II clinical trial assessing the safety and effectiveness of rAAV2tYF–GRK1–RPGR in participants with XLRP due to RPGR mutations (ClinicalTrials.gov Identifier: NCT03316560) and a phase III study assessing the safety and effectiveness of AAV5–hRKp.RPGR for treating Japanese XLRP patients with RPGR mutations (ClinicalTrials.gov Identifier: NCT05926583).

Gene therapy offers the possibility of reversing damage, recovering eyesight, and transforming the management of RP. As our understanding of the molecular mechanisms beneath RP improves, novel therapeutic avenues may emerge.

#### 4.1.3 Age‐related macular degeneration

Macular degeneration, often called AMD, is the primary contributor of severe, permanent vision loss in adults over 55.^117^ AMD manifests in two primary forms: dry AMD and neovascular AMD.^118^ Genetic factors are crucial players in AMD pathogenesis, with 103 AMD‐related genes or loci identified thus far.^119^ Notably, changes in CFH and HTRA1 loci are significant contributors to AMD.^120^, ^121^ Additionally, genes related to lipid metabolism, such as ApoE,^122^ tissue inhibitor of metalloproteinases‐3 (TIMP3),^123^ and hepatic lipase,^124^ have been implicated in the disease. Current clinical treatments for AMD include laser and radiotherapy,^125^ photodynamic therapy with ranibizumab for polypoidal choroidal vasculopathy,^126^ and anti‐VEGF therapy, the primary therapeutic approach for neovascular AMD. The principal pharmacological agents utilized in anti‐VEGF therapy include anti‐VEGF monoclonal antibody fragments pegaptanib^127^ and ranibizumab,^128^ fusion VEGF binding proteins aflibercept^129^ and conbercept,^130^ and bevacizumab.

Gene therapy has demonstrated promise in AMD therapy. Research has indicated the long‐term safety and effectiveness of AAV2 vector‐mediated VEGF therapy.^131^ However, severe adverse events like atrial fibrillation, retinal detachment, and visual impairment in all doses, with incidence rates from 16.67 to 33.33% in different dose groups, happened in a phase I/II trial ascertaining the safety and tolerability of RGX‐314, a rAAV vector containing coding sequences for soluble anti‐VEGF after 24 weeks of injection (ClinicalTrials.gov Identifier: NCT03066258). Another phase I trial assessing the safety and tolerability of a single intravitreal administration of AAV2‐sFLT01 revealed no severe eye‐related adverse events (ClinicalTrials.gov Identifier: NCT01024998), suggesting that the tactic is safe and well tolerated across all dosage levels.^132^

ADVM‐022, developed by Adverum Biotechnologies, is presently undergoing a phase I clinical trial as an intravitreal gene therapy for neovascular AMD. Preliminary results have confirmed its safety and efficacy in maintaining sustained levels of aflibercept, suggesting a potential reduction in treatment burden and improvement in patient vision outcomes.^133^ Suprachoroidal injections of RGX‐314, an AAV8 vector expressing anti‐VEGF‐A Fab, have shown promising results in preclinical studies, suppressing VEGF‐mediated vasodilation and vascular leakage in rats.^134^ Another potential therapy with BT2, a dibenzoxazepinone, has exhibited inhibitory effects on vascular permeability and angiogenesis via suppressing retinal CD31, phospho‐extracellular signal‐regulated kinase, vascular cell adhesion molecule‐1, and VEGF‐A_165_ expression in neovascular AMD.^135^ Additionally, human complement factor I carried by AAV constructs is successfully expressed in the retina of C57BL/6J mice, demonstrating functional activity of the secreted proteins in vitreous humor.^136^ HMR59, an AAV2 vector expressing sCD59, is presently under scrutiny in two phase I clinical trials: HMR‐1002 (NCT03585556) for neovascular AMD (ClinicalTrials.gov Identifier: NCT03585556) and HMR‐1001 (NCT03144999) for dry AMD (ClinicalTrials.gov Identifier: NCT03144999). Moreover, overexpression of AAV‐mediated β‐site amyloid precursor protein cleaving enzyme (BACE1) in the RPE has shown promising results, preventing retinal function loss and retinal degeneration for up to 6 months.^137^ Two phase III clinical trials are ongoing to examine the safety of intravitreal injection of Zimura (Complement C5 Inhibitor) in geographic atrophy patients (ClinicalTrials.gov Identifier: NCT04435366 and NCT05536297).

In summary, expanding therapeutic targets beyond VEGF‐A is an encouraging tactic for addressing the pathogenesis and clinical manifestation of neovascular AMD. Targeting various pathways may enhance treatment response, reduce resistance, and pave the way for future specialized therapies for neovascular and dry AMD.

#### 4.1.4 Treatment of other ocular degeneration disorders

Stargardt disease is an autosomal recessive disease featured by macular degeneration and progressive visual impairment, primarily attributed to ABCA4 gene mutations.^138^ A dual AAV vector split‐intein adenine base‐editing approach is able to rectify the most prevalent mutation in ABCA4 (c.5882G>A, p.G1961E) in human retinal organoid and mutation‐carrying mice. This method sets the stage for accurate and effective gene editing in other neurodegenerative ocular diseases.^139^ A phase II clinical trial initiated in 2022 is investigating vMCO‐010 optogenetic therapy, an AAV2‐based vector, for Stargardt disease (ClinicalTrials.gov Identifier: NCT05417126). In a previous phase I/II trial (ClinicalTrials.gov Identifier: NCT01469832), dose‐dependent subretinal hyperpigmentation was observed in all patients post sub‐retinal transplantation, persisting beyond systemic immunosuppression. Marginal, often transient, enhancements in best‐corrected visual acuity (BCVA) were noted in four subjects. However, no overall benefit was detected at 12 months, with potential harm suggested in one high‐dose case.^140^

Enhanced S‐cone syndrome is characterized by decreased rod photoreceptors, excessive proliferation of S‐cones, and variable disruptions in M‐ and L‐cone development. The transcription factor Nuclear Receptor Subfamily 2 Group E Member 3 (NR2E3), which governs ultimate differentiation and maturation of rod photoreceptors, is commonly implicated as a causative mutant gene.^141^

Mutations in the retinoschisis 1 (RS1) gene are connected to X‐linked juvenile retinoschisis (XLRS), a degenerative retinopathy.^142^ A phase I/II clinical trial (ClinicalTrials.gov: NCT02317887) evaluating AAV8‐RS1 gene therapy for XLRS has demonstrated that intravitreal administration of AAV8‐RS1 results in systemic immune activation, evidenced by dose‐dependent elevation of activated lymphocytes, macrophages, proinflammatory cytokines, and inflammation.^143^, ^144^ A phase I/II study assessing the safety and effectiveness of a rAAV expressing retinoschisis (rAAV2tYF‐CB‐hRS1) in 27 participants with XLRS revealed severe adverse events, including atrial fibrillation, cerebellar stroke, and pulmonary embolism in one out of six patients at the dose of 1 × 10^11^, retinal detachment in one out eight participants at the dose of 3 × 10^11^, retinal detachment in one out of 13 participants at the dose of 6 × 10^11^. Other mild adverse events, including anterior chamber inflammation and iridocyclitis, were also observed in more than half of the participants (ClinicalTrials.gov Identifier: NCT02416622).

Bietti crystalline dystrophy (BCD) is an autosomal recessive inherited retinal disease characterized by chorioretinal degeneration. This condition is triggered by mutations in the cytochrome P450 family 4 subfamily V polypeptide 2 (CYP4V2) gene. BCD is distinguished by the appearance of yellow‐white crystals, variable involvement of the cornea, and complex lipid deposits in the retina.^145^ Several clinical trials are currently initiated, including a phase I/II trial evaluating the safety and effectiveness of ZVS101e (rAAV2tYF‐CB‐hRS1) administered by subretinal injection in BCD patients (ClinicalTrials.gov Identifier: NCT05832684), a multicenter study evaluating the safety and tolerability of a single subretinal application of VGR‐R01, a novel AAV expressing human CYP4V2 (ClinicalTrials.gov Identifier: NCT05694598), and a phase I clinical study ascertaining the safety of rAAV2/8‐hCYP4V2 gene replacement therapy administered as a single subretinal application in BCD patients (ClinicalTrials.gov Identifier: NCT04722107).

Choroideremia, a monogenic X‐linked chorioretinal dystrophy, manifests as a progressive degeneration of RPE, choroid, and retina caused by mutations in the CHM gene, which encodes Rab escort protein 1 (RPE1), a ubiquitously expressed protein critical for Rab protein prenylation. REP1 is pivotal in intracellular vesicle trafficking, ensuring proper cellular transport mechanisms.^146^ The first phase I/II clinical trial (ClinicalTrials.gov ref. NCT01461213) for CHM commenced in 2011, employing the AAV2/2 expressing REP1 in 14 CHM patients via subretinal administration.^147^, ^148^ A subsequent phase I/II clinical trial subretinally delivering AAV2‐hCHM to the macula in patients with choroideremia revealed no differences in visual acuity between the injected and noninjected eyes at the 2‐year postsurgery mark.^149^ An ongoing phase I clinical trial is exploring the intravitreal application of an AAV capsid variant containing a transgene encoding a codon‐optimized CHM gene (4D‐110) (ClinicalTrials.gov ref. NCT04483440). Despite these efforts, gene therapy for choroideremia treatment still requires further preclinical investigations.

Leber hereditary optic neuropathy (LHON) is the primary cause of bilateral central vision loss among optic neuropathies..^150^ This condition is primarily linked to major alterations in the mitochondrial genes ND1, ND4, and ND6, leading to increased oxidative stress within the optic nerve cells and subsequent nerve cell damage.^151^ A series of phase III clinical trials with ClinicalTrials.gov identifier numbers NCT02652780 (REVERSE), NCT02652767 (RESCUE), NCT03406104 (RESTORE), NCT03293524 (REFLECT), and NCT03295071 (REALITY) have been conducted to examine the effectiveness of a single intravitreal administration of GS010 to enhance retina functions and structures in individuals with LHON caused by G11778A mutation in ND4. The treated individuals exhibited a clinically relevant and prolonged progress in their visual acuity compared with natural progression of the disease.^152^ Another phase I trial assessed the safety of scAAV2‐P1ND4v2 for LHON patients due to G11778A mutation in mitochondrial DNA (ClinicalTrials.gov Identifier: NCT02161380). The results indicated a beneficial safety and tolerability, with dosage‐associated incidents of uveitis being the mere investigational product‐associated adverse event. Some treated and fellow eyes in the chronic and acute bilateral groups exhibited betterment of ≥15‐letter BCVA, whereas all study eyes (BCVA ≥ 20/40) in the unilateral acute group experienced a loss of ≥15 letters over 1 year despite treatment.^153^ Overall, larger, randomized controlled trials are necessary to examine the safety and effectiveness of gene therapy for LHON.

### 4.2 Neurological diseases

#### 4.2.1 Spinal muscular atrophy

SMA is a severe degenerative condition featured by progressive motor neuron deterioration.^154^ This autosomal recessive disease is caused by a mutation in the SMN1 gene responsible for motor neuron survival and occurs in approximately one in 10,000 live births. SMA type I, the most severe form, affects approximately 60% of those diagnosed.^155^ Several therapeutic approaches are available for SMA, with effective strategies focused on enhancing SMN production through modifying SMN2 splicing or faulty SMN1 gene replacement.^156^, ^157^ The most successful methods include viral gene therapy for defective SMN1 gene replacement and using antisense oligonucleotides or small molecules for SMN2 splicing manipulation, thereby increasing functional SMA protein production.^158^

The compact nature of the SMN1 gene renders it amenable to delivery via AAVs, particularly AAV9, renowned for its capacity to penetrate the blood–brain barrier, thus serving as an optimal delivery vector. AVXS‐101, a dsDNA, transports one or multiple copies of SMN1 to specific motor neurons’ nuclei, facilitating sustained gene expression.^159^ A phase I clinical trial executed in 2017 examined the safety of intravenous administration of AAV9‐SMN1 (AVXS‐101) in infants with SMA type I. Three infants were given AVXS‐101 at a lower dose and 12 received a higher dose as a single intravenous infusion. Although AAV‐induced liver injury was observed in some cases, the treatment was generally well tolerated following prednisolone management. In the interim analysis conducted when the patients were between 20 and 32 months old, 11 infants in the higher dose group could sit unaided, nine could roll over, and two could walk independently.^160^

These innovative therapies are expanding our comprehension of SMA's biology and pathogenesis, offering hope for disease reversal or reduction, especially with early intervention. By targeting different aspects of SMN deficiency, these treatments will provide valuable insights into SMN's roles, ultimately clarifying the disease's pathogenesis. Thus far, overexpression with this gene transfer approach has not shown harmful effects in preclinical models.

#### 4.2.2 Duchenne muscular dystrophy

Duchenne muscular dystrophy (DMD) is a degenerative muscle disease triggered by DMD gene mutations, causing dystrophin protein loss.^161^ It manifests as muscle weakness, mobility loss by ages 9−14 years, and life‐threatening cardio‐respiratory complications.^162^ Due to its extensive size, the DMD gene is prone to spontaneous mutations, posing challenges for gene therapy. However, researchers are developing strategies to miniaturize the gene, inspired by a Becker muscular dystrophy patient who retained mobility despite a significant gene deletion. Current gene therapy trials by Sarepta Therapeutics, Pfizer, and Solid Biosciences are testing transgenes with varying spectrin repeats and hinges for safety and efficacy.^83^ A nonrandomized controlled trial performed at Nationwide Children's Hospital in Columbus, Ohio assessed the safety and efficacy of intravenous administration of rAAVrh74.MHCK7.micro‐dystrophin in DMD patients over 1 year. The findings indicate that this therapy may yield superior functional improvement compared with standard care^163^ (ClinicalTrials.gov Identifier: NCT03375164). Delandistrogene moxeparvovec (SRP‐9001) is a gene transfer vector expressing a truncated dystrophin protein. In a two‐part, double‐blind phase II study, this therapy was evaluated in aged 4–8‐years‐old DMD patients, with primary endpoints being changes in dystrophin expression and North Star Ambulatory Assessment score. Patients were randomized to administrate either a placebo or the therapy. The results demonstrated significant SRP‐9001 dystrophin expression in all patients, with an average increase from the starting point to week 12 being 23.82% in Part 1 and 39.64% in Part 2. These findings suggest strong SRP‐9001 dystrophin expression and NSAA stabilization after maximum 2 years of treatment^164^ (ClinicalTrials.gov Identifier: NCT03769116). However, platelet transfusion and eculizumab treatment were necessary for a different participant with a condition resembling atypical hemolytic uremic syndrome caused thrombocytopenia. Pfizer has disclosed plans for a randomized, multicenter, double‐blind, and placebo‐controlled phase III trial involving 99 participants (C3391003) (ClinicalTrials.gov identifier: NCT04281485). Solid Biosciences launched the IGNITE DMD study, a phase I/II open‐label clinical trial (ClinicalTrials.gov identifier: NCT03368742) using SGT‐001, an AAV9 vector expressing SRs 16/17‐encoded neuronal nitric oxide synthase (nNOS)‐binding domain under the control of a CK8 muscle‐specific promoter, aiming to improve perfusion in skeletal and cardiac muscles. Till now, six participants have been enrolled and treated, with three receiving a low dose (5.0 × 10^13^ vg/kg) and three receiving a high dose (2.0 × 10^14^ vg/kg). However, recurring issues with complement activation have led to two clinical holds by the US FDA.^165^

Replacing the mutated DMD gene with a normal one theoretically holds the potential to cure the disease. However, the gene's large size and the muscle's widespread distribution pose significant challenges. To overcome these hurdles, researchers have developed a condensed micro‐dystrophin gene and a systemic gene transfer method using AAV. Animal model investigations have shown improved muscle strength and reduced dystrophic cardiomyopathy. Several clinical trials aim to examine its safety and tolerability in DMD patients. Although these trials will not conclusively prove clinical efficacy, they will offer valuable insights into the potential of synthetic micro‐dystrophin AAV vectors for whole‐body DMD treatment.

#### 4.2.3 X‐linked myotubular myopathy

X‐linked myotubular myopathy (XLMTM) is a genetic disease due to mutations in the myotubularin 1 (MTM1) gene located at Xq28.^166^ MTM1 encodes myotubularin, a ubiquitously expressed phosphoinositide phosphatase critical for skeletal muscle development and maintenance.^167^ Its loss‐of‐function mutations disrupt the excitation‐coupling contraction mechanisms and T‐tubule network organization, particularly evident in severe XLMTM, the most prevalent form presenting symptoms at birth. These symptoms include hypotonia, external ophthalmoplegia skeletal muscle weakness, and respiratory insufficiency.^168^ A comprehensive international, prospective, longitudinal, natural history study involving 45 male participants aged 3.5 months to 56.8 years showed differentiation of three patient groups in motor function measure 32 total scores, grip and pinch strengths, and various respiratory measures. Some patients experienced motor milestone loss, whereas longitudinal data revealed a 2% decrease in motor function measured 32 total scores, indicating slow progression in male survivors regardless of phenotype. Additionally, 26% of patients exhibited detectable anti‐AAV8 neutralizing activity.^166^ In a study involving young patients with XLMTM, AT132 gene therapy showed promising outcomes. Treated patients demonstrated significant improvements in neuromuscular and respiratory functions, achieving key motor milestones and reducing ventilator dependency. Positive histopathological changes were observed in muscle biopsies. Despite some adverse events, the therapy's safety profile was manageable. However, following a patient's death, the US FDA suspended the study pending further investigation into the cause, emphasizing the critical importance of ongoing safety monitoring for AT132.^169^ Although substantial advancement in systemic gene delivery has been made, challenges persist, necessitating thorough analysis and long‐term surveillance of therapy effectiveness and safety. The severe complications observed in the XLMTM trial underscore the need for continued investigation and refinement of gene therapy approaches.

### 4.3 Central nervous system

Gene therapy has significantly progressed in addressing neurodegenerative disorders in recent decades, driven by a deeper understanding of their underlying pathogenic mechanisms. This enhanced understanding has not only facilitated unveiling new therapeutic targets and vectors but also enabled precise targeting of the root causes of these disorders.^170^ One of the most profound advantages of gene therapy lies in its ability to address complex physiological barriers in organs such as the eye, cochlea, and central nervous system (CNS). These barriers, including the blood–cerebrospinal fluid, blood–retina, and blood–brain barriers, have long posed challenges to effective treatment.^21^, ^171^ However, gene therapy offers a promising avenue for surmounting these obstacles. Moreover, gene therapy can potentially manage genetic targets that are resistant to traditional treatments. By modulating gene expression, either by silencing or overexpressing, gene therapy can effectively address various types of mutations, offering hope for patients with otherwise untreatable conditions.

#### 4.3.1 Alzheimer's disease

Alzheimer's disease is a neurodegenerative disorder pathologically defined as significant neuronal loss and the accumulation of intracellular neurofibrillary tangles and extracellular amyloid plaques in the brain.^172^ In early‐onset, three specific genes have been identified as causative factors: amyloid precursor protein located on chromosome 21, presenilin 1 on chromosome 14, and presenilin 2 on chromosome 1.^172^ They harbor over 300 mutations, leading to elevated levels of overall β‐amyloid (Aβ), increased Aβ42/40 ratios, and/or the formation of Aβ polymers.^172^ Studies have investigated the potential of nerve growth factor (NGF), a protein that could potentially restore and protect neuron functions in Alzheimer's disease. However, effectively delivering NGF has posed challenges. In an open‐label clinical trial, gene transfer and stereotactic surgery were employed to administer NGF. Ten Alzheimer's disease patients received bilateral injections of a genetically engineered gene therapy vector (AAV2‐NGF, CERE‐110) into a specific brain region. The strategy was deemed safe and well tolerated over 2 years, without accelerated decline. Autopsies confirmed long‐term, targeted NGF expression and activity, supporting the approach's feasibility and paving the way for a larger, double‐blind, sham‐operation‐controlled trial.^173^ Several other clinical trials are ongoing to address Alzheimer's disease, including a phase I trial examining the safety and tolerability of Libella gene therapy AAV‐hTERT (ClinicalTrials.gov Identifier: NCT04133454), a phase I trial examining whether continuously delivering brain‐derived neurotrophic factor (BDNF) into the brain by AAV2‐BDNF can slow or prevent cell loss in individuals with Alzheimer's disease and mild cognitive impairment (ClinicalTrials.gov Identifier: NCT05040217), and a study evaluating the safety and potential toxicity of directly administering AAVrh.10hAPOE2 (LX1001) gene transfer vector expressing human apolipoprotein E2 (APOE2) into APOE4 homozygotes with Alzheimer's disease (ClinicalTrials.gov Identifier: NCT03634007). Despite initial setbacks, gene therapy is continuously investigated for treating Alzheimer's disease.

#### 4.3.2 Parkinson's disease

Parkinson's disease is a neurodegenerative disease featured by slow movement, walking difficulties, and eventual cognitive decline due to dopamine‐producing neuron loss in the basal ganglia of the brain. Gene therapy approaches have been explored to address Parkinson's disease. Preliminary clinical trials have explored gene therapy strategies using viruses to modify GABAergic neuronal signaling, such as AAV2‐GAD (AAV‐borne glutamic acid decarboxylase).^174^ In a phase I trial involving the transfer of the AAV‐GAD gene into the subthalamic nucleus, the procedure was deemed safe and well tolerated^175^ (ClinicalTrials.gov Identifier: NCT00195143). Currently, Brain Neurotherapy Bio, Inc. is conducting a nonrandomized, open‐label safety trial to explore the usage of AAV2‐GDNF (AAV2‐borne glial cell line‐derived neurotrophic factor) for managing Parkinson's disease. In this phase I trial, AAV2‐GDNF is delivered into the putamen. The trial is still in the participant recruitment stage, and no data have been published yet (ClinicalTrials.gov Identifier: NCT04167540). However, a phase II study of AAV2‐neurturin (CERE‐120) demonstrated no obvious difference in motor skill enhancement between the treatment and control groups. Serious side effects were noted in both groups, with a higher incidence in the AAV2‐neurturin group. Additionally, a few patients in both groups developed tumors. Consequently, the study concluded that AAV2‐neurturin treatment is not more effective than the placebo in improving motor skills over a year.^176^ In gene therapy, precisely delivering target genes into a specific brain area using an appropriate vector is crucial. This process carries inherent risks, including disrupted walking patterns, damage to the dorsal root ganglia, loss of muscle coordination (ataxia), and elevated transaminase levels.^177^

#### 4.3.3 Canavan disease

Canavan disease is a type of leukodystrophy arising from harmful aspartoacylase (ASPA) gene mutations. ASPA, produced by oligodendrocytes, is crucial for breaking down N‐acetylaspartate (NAA) via deacetylation. Increased NAA levels in the CNS can lead to various consequences, including abnormal myelination, parenchymal edema (swelling of brain tissue), and vacuolation (formation of small cavities in the white matter).^178^ A phase I/II clinical trial is undergoing to assess the safety, tolerability, and pharmacodynamic activity of AAV9 BBP‐812 in children affected by Canavan disease. AAV9 BBP‐812 is a specially engineered vector carrying the ASPA gene, controlled by a universal promoter, aimed to reinstate ASPA expression in both neuronal and non‐neuronal cell types (ClinicalTrials.gov Identifier: NCT04998396). A phase I/II clinical trial has been launched to examine the safety, pharmacodynamics, and effectiveness of a single intracerebroventricular dose of rAAV‐Olig001‐ASPA in up to 24 pediatric patients diagnosed with Canavan Disease. This trial represents the first‐in‐human protocol designed to evaluate the neurosurgical administration of a novel gene therapy vector targeting oligodendrocytes (ClinicalTrials.gov Identifier: NCT04833907).

#### 4.3.4 Aromatic l‐amino acid decarboxylase deficiency

Aromatic l‐amino acid decarboxylase (AADC) deficiency disorder is a rare genetic condition stemming from specific variations in the dopa decarboxylase gene on chromosome 7, impacting neurotransmitter synthesis. Although symptom severity varies, approximately 80% of patients have a severe form, typically manifesting symptoms from infancy, including hypotonia, growth retardation, and significant motor impairments.^179^ Chien et al.^180^ conducted a trial assessing AAV2‐hAADC gene therapy in patients with AADC deficiency, with 10 patients receiving the treatment. The results showed improvement in motor function and an increase in homovanillic acid concentrations, with one patient died from an unrelated cause. Reported adverse events were generally mild, with transient dyskinesia resolved with risperidone. This study highlights the possible effectiveness and tolerability of AAV2‐hAADC gene therapy for AADC deficiency^180^ (ClinicalTrials.gov Identifier: NCT01395641). In three separate clinical trials, children with AADC deficiency administrated eladocagene exuparvovec, an AAV2‐mediated gene therapy, through bilateral intraputaminal infusions. The safety group consisted of 26 patients, whereas the treatment group included 21 patients. After treatment, average body weight increased, and the frequency of oculogyric crises improved. Although dyskinesia occurred as an adverse event, it is generally resolved with standard pharmacotherapy. Overall, eladocagene exuparvovec gene therapy showed positive effects on body weight, oculogyric crises, and dyskinesia in children with AADC deficiency.^181^ In another study involving 26 patients, eladocagene exuparvovec gene therapy showed significant and long‐lasting improvements in motor and cognitive abilities for patients with AADC deficiency. Increased dopamine production, symptom relief, and improved quality of life were observed, with a favorable safety profile and manageable side effects^182^ (ClinicalTrials.gov Identifier: NCT01395641, NCT02926066). Looking ahead, research endeavors can explore noninvasive viral vector delivery or investigate alternative emerging treatments. Such studies hold promise for providing benefits to patients with AADC.

### 4.4 Hematological diseases

Hemophilia A and B are genetic bleeding diseases due to deficiencies or dysfunctions of blood coagulation factors VIII or IX, respectively. Hemophilia A impacts about one in 5000 individuals, whereas hemophilia B affects one in 25,000 live male births. Severe cases exhibit <1% (<1 IU/dL) of factor VIII or IX activity, leading to recurrent spontaneous bleeding in muscles, soft tissues, and critical areas like the brain, as well as excessive bleeding during surgeries or injuries.^183^ Despite the expanding range of treatments available for hemophilia, gene therapy presents a particularly promising option, potentially offering a cure by enabling the body's own production of factors VIII or IX following delivering a functional gene.^184^

#### 4.4.1 Hemophilia A

Individuals diagnosed with hemophilia A depend on exogenous administration of factor VIII to mitigate bleeding episodes in joints, soft tissues, and CNS. Although gene therapy is effective in treating hemophilia B patients, the extensive size of the factor VIII coding region has posed challenges for achieving comparable outcomes in individuals with hemophilia A through gene therapy. Rangarajan et al.^185^ intravenously administered a single dose of a codon‐optimized AAV5 vector encoding a B‐domain‐deleted human factor VIII (AAV5‐hFVIII‐SQ) in nine men with severe hemophilia A. Participants in the high‐dose group achieved prolonged factor VIII activity > 50 IU/dL, leading to reduced bleeding events and elimination of the need for exogenous factor VIII. The main side effect was a slight augment in the serum alanine aminotransferase level. The only severe adverse event was preexisting chronic joint disease progression in one participant^185^ (ClinicalTrials.gov Identifier: NCT02576795). In a longitudinal study, a cohort of 15 adults with severe hemophilia A (factor VIII level ≤1 IU/dL) who had previously administrated a single infusion of AAV5‐hFVIII‐SQ at different dosages were followed up for 3 years. The findings demonstrated that gene therapy utilizing the AAV5‐hFVIII‐SQ vector provided a durable and clinically significant improvement in individuals with hemophilia A^186^ (ClinicalTrials.gov Identifier: NCT02576795). A phase III open‐label, single‐arm, multicenter trial assessed BMN 270, an AAV5 with a FVIII gene, in 134 patients with severe hemophilia A at a dose of 6 × 10^13^ vg/kg. This trial represented the largest gene therapy trial conducted on hemophiliac patients. The safety and efficacy results indicated that 132 individuals who were negative for the human immunodeficiency virus had mean FVIII activity of 41.9 IU/dL between 49 and 52 weeks. This was associated with a 98.6 and 83.8% decrease in treated bleeding and the use of FVIII concentrate, respectively. Transaminitis occurred in 85.8% of the subjects and was treated with temporary immunosuppression. No thromboembolic events, cancer, or use of FVIII inhibitors were reported. The initial observations appear promising^187^ (ClinicalTrials.gov Identifier: NCT03370913). In a phase I/II trial, 18 men with hemophilia A were administered an AAV vector (SPK‐8011) to promote factor VIII expression. Among the participants, 16 maintained stable factor VIII activity for more than 2 years, showing a remarkable 91.5% reduction in bleeding episodes. No significant safety issues were identified. The study has progressed to phase III evaluation, aiming to gather additional data on the efficacy and safety of this approach^188^ (ClinicalTrials.gov Identifier: NCT03003533 and NCT03432520). In another phase I/II trial, B‐domain‐deleted FVIII codon‐optimized cDNAs (BAY 2599023, AAVhu37.hFVIIIco) were delivered via an AAVhu37 capsid under the control of a liver‐specific promoter/enhancer element. Nine individuals have been progressively included into one of the three dose cohorts (0.5 × 10^13^, 1 × 10^13^, and 2 × 10^13^ vg/kg) thus far. BAY 2599023 was delivered to six patients at 0.5, 1.0, and 2.0 × 10^13^ GC/kg dosages. All treated individuals showed symptoms of efficient blood coagulation and measurable, stable expression of endogenous Factor VIII (FVIII), demonstrating a successful proof‐of‐concept for the treatment^189^ (ClinicalTrials.gov Identifier: NCT03588299).

#### 4.4.2 Hemophilia B

Hemophilia B, a rare genetic disease linked to the X chromosome, is characterized by mutations in the F9 gene, responsible for producing blood coagulation factor IX, also known as the Christmas factor. Factor IX deficiency results in prolonged bleeding, which can occur spontaneously or following an injury. Hemophilia B predominantly affects males, with females mainly acting as carriers, resulting in a global incidence of about one in 30,000 male births.^190^ In a clinical study, 10 patients with severe hemophilia B were systemic administrated varying dosages of scAAV2/8‐LP1‐hFIXco gene therapy drug via a single intravenous infusion. Their factor IX expression and activity were stable for over 8 years after the treatment, leading to a dramatic decrease in annual FIX concentrate usage and bleed rate. Although no late toxicities were observed, most patients had persistently high levels of anti‐AAV8 capsid‐specific antibodies. The study suggests that reducing the capsid load does not necessarily reduce hepatotoxicity in severe hemophilia B patients, indicating the involvement of other factors in this process^191^ (ClinicalTrials.gov Identifier: NCT00979238). In a phase III study, 54 men with hemophilia B received a single infusion of etranacogene dezaparvovec, regardless of any preexisting antibodies. The treatment was beneficial and safe for patients with predose AAV5 neutralizing antibody titers < 700, and no serious adverse events related to the treatment were reported. The use of factor IX concentrate also significantly decreased after treatment^192^ (ClinicalTrials.gov Identifier: NCT03569891). In a multicenter, open‐label phase I/II clinical trial, investigators evaluated the safety and effectiveness of FLT180a in patients with severe or moderately severe hemophilia B, featured by factor IX levels ≤2% of the normal value. Although one patient with high factor IX level demonstrated severe arteriovenous fistula thrombosis, patients treated with low doses of FLT180a had normal factor IX levels over time when concurrently given immunosuppressant glucocorticoids, with or without tacrolimus^193^ (ClinicalTrials.gov Identifier: NCT03369444, NCT03641703).

Ongoing research on modifications of AAV vectors to enhance transgene delivery and expression, along with initial successes in genome editing in animal models, highlight the need to continue developing “best in class” solutions for treating hemophilia B. The diverse AAV gene therapy trials present valuable alternatives for the hemophilia community, enabling the treatment of patients with existing antibodies against one serotype using alternative serotypes. Unlike hemophilia B, higher vector load are required to address hemophilia A. In addition, FVIII expression in subjects with hemophilia A seems to diminish gradually after treatment compared with patients with hemophilia B.

### 4.5 Cardiovascular diseases

Chronic heart failure is a progressive cardiovascular disease featured by heart's failure to provide sufficient blood for metabolism or handle increased blood returning to the heart. The diseases often present as symptoms like shortness of breath, difficulty breathing when lying flat, ankle swelling, and feelings of tiredness and weakness.^194^ Multiple factors contribute to heart failure, with the most prevalent causes being coronary artery disease, high blood pressure, and heart valve diseases.^195^ Coronary artery disease is a chronic condition featured by inflammation and fat accumulation in the inner and middle layers of the coronary arteries, forming blockages known as atherosclerotic plaques. These plaques narrow or even completely obstruct the arteries, decreasing blood flow, causing oxygen and nutrient deficiencies, and leading to ischemia.^196^ Gene therapy for heart conditions can be administered through direct injection into the heart muscle or via infusions into the blood vessels. The injection may involve piercing the chest wall or using less invasive catheter‐based methods. Blood vessel infusions can be performed using either an antegrade method, where the therapy is infused forward through the coronary arteries, or a retrograde method, where the therapy is infused backward through the coronary veins. However, it is necessary to note that some of these techniques are currently only utilized in experimental animal studies.^197^

#### 4.5.1 Heart failure

CUPID 2 trial, the largest gene transfer study on heart failure patients, concluded that AAV1/SERCA2a gene therapy did not improve primary outcomes compared with placebo, despite its safety. This outcome underscores the importance of further research into gene therapy for heart failure treatment^198^ (ClinicalTrials.gov Identifier: NCT01643330). An analysis of explanted hearts after SERCA2a therapy demonstrated a diminished quantity of vector‐derived DNA compared with preclinical studies, implying that inefficient delivery mechanisms could have played a role in the unsuccessful outcomes.^199^ Based on these findings, the ongoing MUSIC‐HFrEF1 trial is investigating whether a higher AAV dose could enhance vector uptake and improve efficacy. This phase I/II study includes a randomized, double‐blind, placebo‐controlled phase II segment involving 56 subjects with advanced heart failure (ClinicalTrials.gov Identifier: NCT04703842). In another phase II clinical trial (ClinicalTrials.gov Identifier: NCT00787059) in US medical centers (randomization occurred from July 19, 2010, to October 30, 2014), 56 individuals received either intracoronary administration of Ad5.hAC6 or placebo. The trial assessed primary outcomes such as duration and ejection fraction at different time points. Although no dramatic difference in exercise duration was noticed between the two groups, participants receiving the highest Ad5.hAC6 dose showed enhanced ejection fractions at 4 weeks but not at 12 weeks. AC6 gene transfer also boosted basal left ventricular peak but not arrhythmias. Notably, the hospital admission rate due to heart failure was lessened in the therapy group than the placebo group. The study concluded that AC6 gene transfer safely improved left ventricular function beyond standard therapy with a one‐time application, suggesting larger trials are needed.^200^ The phase III randomized clinical trial FLOURISH, supported by Renova Therapeutics and involving 536 patients, further investigated this approach. However, in 2019, the trial was retracted from ClinicalTrials.gov to reassess the clinical plan and delivery strategy (ClinicalTrials.gov Identifier: NCT03360448). Currently, a phase I multicenter, open‐label trial is ongoing to examine the safety, feasibility, and effectiveness of BNP116.sc‐CMV.I1c in New York Heart Association Class III heart failure patients. Up to 12 subjects will receive a single intracoronary infusion, followed by 12 months of posttreatment follow‐up and subsequent semi‐structured telephone questionnaires every 6 months for 24 months (ClinicalTrials.gov Identifier: NCT04179643).

#### 4.5.2 Coronary artery disease

Coronary artery disease stands as the foremost cause of mortality and the predominant cardiovascular a disease globally.^201^ Gene therapy with members of the VEGF family emerges as a promising avenue for relieving symptoms in patients with refractory angina.^202^ The therapeutic approach aims to stimulate new blood vessel formation and widen existing vessels by enhancing VEGF expression. This can be accomplished using genetically modified vectors, like VEGF‐carrying adenoviruses. Upon introduction into the ischemic yet viable heart muscle, these vectors can trigger both angiogenesis and arteriogenesis.^203^ The phase I KAT301 trial was the inaugural exploration evaluating the safety and effectiveness of gene therapy using adenovirus‐mediated VEGF‐D^ΔNΔC^ (AdVEGF‐D) delivered directly into the heart muscle of patients with refractory angina^204^ (ClinicalTrials.gov Identifier: NCT01002430). The trial revealed that regions treated with AdVEGF‐D injections exhibited an enhanced blood flow reserve in the heart muscle, as measured by ^15^O‐H_2_O positron emission tomography. Furthermore, the treatment mitigated severe chest pain and improved overall quality of life and was deemed safe and well tolerated without severe associated side effects during the 1‐year follow‐up period^205^ (ClinicalTrials.gov Identifier: NCT01002430). Another clinical study examined the safety and preliminary effectiveness of encoberminogene rezmadenovec (XC001), a new therapy for refractory angina. In this phase I trial, patients received increasing doses of XC001 to assess its safety and initial effectiveness. The primary outcome was safety, monitored over 6 months. Efficacy was measured through changes in exercise duration, myocardial perfusion deficit, angina class, angina frequency, and quality of life. The findings indicated that the highest planned dose of XC001 was well tolerated and improved all parameters, warranting its advancement to phase II^206^ (ClinicalTrials.gov Identifier: NCT04125732).

### 4.6 Lysosomal storage diseases

LSDs comprise approximately 50 genetic conditions resulting from mutations affecting enzymes crucial for lipid and large molecule breakdown and transport. These mutations cause abnormal accumulation of substances in lysosomes, leading to cell death. Despite the wide range of clinical symptoms across different LSDs, over half are related to nervous system degeneration. Sphingolipidoses, rare inherited disorders falling under LSDs, encompass over 10 different diseases, including GM1‐gangliosidosis, AB variant of GM2‐gangliosidosis, as well as Tay‐Sachs, Sandhoff, Fabry, and Gaucher diseases.^207^ GM1‐gangliosidosis is caused by alterations in the β‐galactosidase 1 (GLB1) gene, resulting in enzyme deficiency and GM1‐ganglioside accumulation, primarily impacting the nervous system.^208^ Three AAV vectors carrying the GLB1 gene are currently in clinical trials: AAV9, AAVhu68, and AAVrh10. Both AAV9 and AAVrh10 are frequently used for nervous system‐related diseases.^207^ In 2019, Zolgensma, an AAV9‐based gene therapy medication, received approval for treating SMA. Intravenous administration of a single dose in affected children successfully transduced motor neurons and sustained motor activity^209^ (ClinicalTrials.gov Identifier: NCT02122952). GM2‐gangliosidosis arises from a deficiency in heterodimeric β‐hexosaminidase A encoded by HEXA and HEXB genes. Mutations in HEXA lead to Tay‐Sachs disease, whereas mutations in HEXB result in Sandhoff disease. Both disorders manifest as GM2‐ganglioside accumulation, causing profound neurodegeneration and neuroinflammation.^210^ A prospective, open‐label phase I/II clinical trial has been created to examine the safety and effectiveness of intrathecal administration of TSHA‐101, an AAV9 viral vector carrying the HEXA and HEXB genes, for infantile‐onset GM2‐gangliosidosis treatment (ClinicalTrials.gov Identifier: NCT04798235). To date, two major advancements have emerged in gene therapy strategies for sphingolipidoses. First, various AAV serotypes are employed to deliver genes encoding deficient proteins via intravenous, intracerebral, intrathecal, or other routes, often combining multiple administration methods for optimal outcomes. Second, a combined cell and gene therapy approach is explored, acknowledging partial disease progression impediment observed with bone marrow transplantation in sphingolipidoses patients. However, insufficient endogenous expression of the defective protein in donor cells remains challenging. Although gene therapy holds promise for sphingolipidoses, further investigation and comprehensive safety assessments are imperative to validate these innovative approaches.^207^

Fabry disease, caused by mutations in the α‐galactosidase A (GLA) gene, leads to enzyme deficiency and globotriaosylceramide accumulation in various cell types. Seven clinical trials are underway investigating several AAVs encoding the GLA gene for Fabry disease treatment, exploring AAV6 (ClinicalTrials.gov Identifier: NCT04046224 and NCT05039866) and novel modified AAV serotypes based on preclinical studies, including AAVS3, a modified AAV8 variant (ClinicalTrials.gov Identifier: NCT04040049 and NCT04455230). Although the copyright holders have not disclosed the second modified AAV serotype, it is referred to as 4D‐C102 in clinical trials (ClinicalTrials.gov Identifier: NCT04519749 and NCT05629559). STAAR, a multicenter, open‐label, dose‐ascertainment study, evaluates the AAV2/6 vector‐based pharmaceutical ST‐920 (ClinicalTrials.gov Identifier: NCT04046224, Sangamo). FLT190, an investigational gene therapy currently undergoing phase I/II clinical trials, is designed based on a platform concurrently developed for treatments of hemophilia A and B as well as Gaucher disease. FLT190 features a codon‐optimized GLA transgene regulated by a liver‐specific promoter enclosed within a synthetic capsid, which enhances transduction efficiency in human hepatocytes compared with other wild‐type AAV serotypes^211^ (ClinicalTrials.gov Identifier: NCT04455230). Another clinical study employing an attenuated AAV (4D‐310, 4D Molecular Therapeutics) is currently in the recruitment phase (ClinicalTrials.gov Identifier: NCT04519749).

Gaucher disease is a genetic disorder featured by unprocessed glucosylceramide accumulation within enlarged macrophages, known as Gaucher cells, due to enzyme deficiency due to by β‐glucocerebrosidase 1 (GBA1) gene mutations, resulting in significant organ damage, particularly affecting the bone marrow, spleen, and liver.^212^ At present, four clinical trials using AAV‐based gene therapy for Gaucher disease are recruiting patients (ClinicalTrials.gov Identifier: NCT04411654, NCT05487599, NCT05324943, NCT04127578). In these studies, AAVs are given intravenously, with three utilizing AAV9 to carry the GBA1 gene.

Although significant progress has been made in clinical investigations, effectively addressing various metabolic phenotypes via AAV‐mediated gene transfer requires intensified efforts to overcome the obstacles hindering successful translation into human therapeutics. Encouragingly, rapid advancements in vector evolution and our comprehension of disease pathophysiology instill confidence that these challenges are surmountable. The pivotal element will be the judicious selection of target disease phenotypes, cognizant of biological, ethical, and logistical considerations, in conjunction with an iterative methodology that integrates preclinical and clinical research.

## 5 CURRENT GENE‐EDITING STRATEGIES IN HUMAN DISEASES

Gene therapy has advanced significantly with the development of various gene‐editing technologies, including EMNs, ZFNs, TALENs, and the CRISPR/Cas9 system. EMNs, proteins engineered to recognize and cleave specific DNA sequences, have been applied in preclinical and early clinical studies for treating genetic disorders. For instance, a phase I trial utilized EMNs to disrupt CCR5 gene, encoding a coreceptor for HIV entry into cells (ClinicalTrials.gov Identifier: NCT00842634). Infusion of ZFN‐modified cells was deemed safe, resulting in a dramatically augmented CD4 T‐cell count and sustained presence of modified cells, suggesting the potential of CCR5‐modified autologous CD4 T‐cell infusions as a safe therapeutic approach for HIV treatment.^213^ ZFNs and TALENs are also utilized in gene therapy. These engineered nucleases bind to specific DNA sequences and induce double‐strand breaks, which can be subsequently repaired by the cell's own machinery, enabling targeted gene modifications. ZFNs have been employed in clinical trials for HIV and hemophilia B. For example, Sangamo Therapeutics conducted a phase I trial employing ZFNs to alter the CCR5 gene in T cells of HIV patients (ClinicalTrials.gov Identifier: NCT01044654). Sangamo's phase 1 trial of SB‐FIX‐1501 involving one participant, the first use of ZFN genome editing in a hemophilia B patient, concluded in 2022. Although the trial reported no adverse effects, factor IX expression was only 1.1%, requiring ongoing replacement therapy. Long‐term follow‐up continues due to the rarity of such in vivo human genome editing cases (ClinicalTrials.gov Identifier: NCT04628871).^214^ Another phase I trial is currently undergoing to evaluate the safety and effectiveness of CRISPR/Cas9‐edited T cells in advanced cancer patients. Autologous T cells collected from three cancer patients were modified using Cas9 RNPs targeting TRAC, TRBC, and PDCD1 genes and further engineered with a lentivirus carrying an HLA‐A2*0201‐restricted TCR specific to cancer antigens NY‐ESO1 and LAGE‐1 to generate engineered T cells, called NYCE. NYCE infusion was well tolerated, demonstrating promising results in this initial trial (ClinicalTrials.gov Identifier: NCT03399448).^215^ Despite challenges such as off‐target effects, delivery issues, and immune responses to the Cas9 proteins, the potential of these gene‐editing technologies in treating various diseases is immense and continues to be explored. Table 3 display a selection of ongoing gene‐editing clinical trials in human diseases.

**TABLE 3:** A selection of ongoing gene‐editing clinical trials in human diseases (*Data sources*—Https://www.clinicaltrials.gov/).

System	Disease	Intervention	Strategy	Phase	ClinicalTrials.gov identifier
Hematological malignances	AIDS	Modified CD34+ cells	CCR5 gene modification by CRISPR/Cas9 strategy	Not applicable	NCT03164135
Immune diseases	X‐linked chronic granulomatous disease		Base‐edited hematopoietic stem and progenitor cells	Phase 1+2	NCT06325709
Eye diseases	RP	ZVS203e	CRISPR/Cas9 gene editing	Early Phase 1	NCT05805007
Eye diseases	AMD	HG202	CRISPR/Cas13 RNA‐editing therapy	Early Phase 1	NCT06031727
Eye diseases	Refractory viral keratitis	BD111	CRISPR/Cas9 mRNA gene editing	Not applicable	NCT04560790
Metabolic diseases	Ornithine transcarbamylase deficiency	ECUR‐506	Gene editing	Phase 1+2	NCT06255782
Neurological diseases	DMD	GEN6050X	Human DMD exon 50 skipping base editing drug	Early Phase 1	NCT06392724
Lysosomal storage diseases	MPS‐I	SB‐913	ZFN therapeutic genome editing	Phase 1+2	NCT03041324
Lysosomal storage diseases	MPS‐II	SB‐318	ZFN therapeutic genome editing	Phase 1+2	NCT02702115
Cardiovascular diseases	Heterozygous familial hypercholesterolemia, atherosclerotic cardiovascular disease, and uncontrolled hypercholesterolemia	VERVE‐101	Base‐editing technology	Phase 1	NCT05398029
Immune system diseases	Relapsed or refractory B‐cell malignancies	CTX112	Anti‐CD19 allogeneic CRISPR–Cas9‐engineered T cells	Phase 1+2	NCT05643742
Hematopoietic diseases	Transfusion‐dependent β‐thalassemia	ST‐400	ZFN	Phase 1+2	NCT03432364

Abbreviations: AIDS, acquired immunodeficiency syndrome; LRP, retinitis pigmentosa; AMD, age‐related macular degeneration; DMD, Duchenne muscular dystrophy; CRISPR/Cas, clustered regularly interspaced short palindromic repeats–CRISPR‐associated proteins; ZFN, zinc‐finger nucleases.

## 6 CONCLUSIONS AND PROSPECTS

Gene therapy has emerged as a promising approach for treating various human diseases. Significant advancements have been made, with numerous clinical trials demonstrating its safety and efficacy in diverse conditions, notably genetic disorders such as Fabry disease, hemophilia, and Gaucher disease. Ongoing clinical trials continue to offer valuable insights into these therapies’ safety and effectiveness. The development of viral vectors, such as AAV and lentivirus, has greatly improved the delivery and targeting of therapeutic genes. Additionally, genome editing techniques, like CRISPR–Cas9, have provided researchers and medical professionals with powerful tools for various medical applications, including direct genome editing and epigenetic gene regulation. However, challenges persist in the field of gene therapy. Long‐term safety, effective delivery systems, and immune response management are crucial areas that require attention. The cargo capacity of rAAV remains a constraint that requires attention. Preclinical and clinical studies have consistently demonstrated that rAAV treatment‐related inflammation, particularly from subretinal and intravitreal injection, appears to be dose dependent.^216^ Fortunately, mild inflammation following AAV delivery can often be mitigated with topical, local, or systemic steroid applications.^217^ Moreover, concerns about off‐target effects and decreased transduction efficiency remain significant in gene therapy.^218^ Ensuring the long‐term safety of gene therapy is paramount, as off‐target impacts, and unforeseen consequences of genetic manipulation are actively researched. Developing efficient and targeted delivery systems, particularly for nonviral vectors, poses a significant challenge. Furthermore, immune responses to the vector or the newly introduced gene can hamper therapy effectiveness and pose potential risks to patients.

Gene therapy is not just limited to monogenic disorders, it is now venturing into the realm of complex diseases such as cancer, cardiovascular diseases, and neurodegenerative disorders. Despite the significant barriers posed by high costs associated with gene therapy, the potential it holds for enhancing the quality of life of individuals with various genetic disorders is substantial. Safer vectors need to be designed, and assays for specific cell types should be developed and tested with various vectors containing various genomic elements. Further investigations are needed to examine the long‐term effectiveness and safety profile of gene therapy. However, these challenges are being met with ongoing and progressive investigations, expanding the scope of clinical applications and fostering a promising outlook for effectively treating various human diseases.

## AUTHOR CONTRIBUTIONS

Fanfei Liu conceived and drafted the manuscript and generated the figures and tables. Ruiting Li completed Tables 1, 2, and 3. Zilin Zhu helped working with figures. Fang Lu and Yang Yang edited and revised the manuscript. All authors have endorsed the final manuscript.

## CONFLICT OF INTEREST STATEMENT

The authors have no conflict of interest to disclose.

## ETHICS STATEMENT

Not applicable.

## Data Availability

Not applicable.
